# MarkerPredict*:* predicting clinically relevant predictive biomarkers with machine learning

**DOI:** 10.1038/s41540-025-00603-0

**Published:** 2025-11-21

**Authors:** Daniel V. Veres, Peter Csermely, Klára Schulc

**Affiliations:** 1 Department of Molecular Biology, Semmelweis University, Budapest, Hungary; 2 Turbine Ltd, Budapest, Hungary; 3 Division of Oncology, Department of Internal Medicine and Oncology, Semmelweis University, Budapest, Hungary

**Keywords:** Cancer, Oncology, Computational science, Molecular biology

## Abstract

Precision oncology relies on predictive biomarkers for selecting targeted cancer therapies. Network-based properties of proteins, together with structural features such as intrinsic disorder, are likely to shape their potential as biomarkers. We therefore designed a hypothesis-generating framework that integrates network motifs and protein disorder to explore their contribution to predictive biomarker discovery. This encouraged us to develop MarkerPredict by using literature evidence-based positive and negative training sets of 880 target-interacting protein pairs total with Random Forest and XGBoost machine learning models on three signalling networks. MarkerPredict classified 3670 target-neighbour pairs with 32 different models achieving a 0.7–0.96 LOOCV accuracy. We defined a Biomarker Probability Score (BPS) as a normalised summative rank of the models. The scores identified 2084 potential predictive biomarkers to targeted cancer therapeutics, 426 was classified as a biomarker by all 4 calculations. We detailed the biomarker potential of LCK and ERK1. This study encourages further validation of the high-ranked predictive biomarkers. The development of the MarkerPredict tool (which is available on GitHub) for predictive biomarker identification may have a significant impact on clinical decision-making in oncology.

## Introduction

With the emergence of personalised medicine, advanced molecular diagnostics gained a substantial role in daily medical practice. Choosing between different expensive targeted therapeutics with often serious side effects is a difficult decision, which is frequently helped by detecting the mutation of the targeted protein as a predictive biomarker. However, the question of therapy resistance cannot always be answered with a yes-or-no answer, so this is where the importance of other predictive biomarkers identifying individuals with a favourable or unfavourable drug response comes into play^1^. The mutational status and expression level of additional proteins on the same pathway may affect the efficacy of the targeted therapeutics, as famous examples show. For instance, BRAF mutations can cause therapy resistance to EGFR inhibitors in colon cancer^2^, and somatic or germline BRCA mutations can show sensitivity to PARP inhibitors in multiple cancer types^3^. These diagnostic steps may detect patients with intrinsic or acquired therapy resistance, thus sparing them from unnecessary side effects and helping them finding the most effective treatment option^4^.

Intrinsically disordered proteins (IDPs) are proteins that have regions without tertiary structures. Their special structure could have contributed to their role as biomarkers in the pathogenesis of certain common diseases, such as neurodegenerative diseases^5–7^, myocardial ischaemia^8^, fibrosis^9^ and amyloidosis^10^. Some IDPs were also discovered to be cancer biomarkers^11–13^, such as CETN1 in prostate and pancreatic cancer^14^. Cancer testis antigens, which are implied to be IDPs themselves such as CETN1, may also be good cancer biomarker candidates^15^. Flexibility of IDPs to establish new connections may contribute to their important role in cancer signalling^16,17^. However, there were no articles directly discussing the role of IDPs as cancer biomarkers at the system level which prompted us to examine the potential use of target-related IDPs as predictive biomarkers of cancer using system-level signalling networks. System-wide coverage of IDPs is especially important, since IDPs are very challenging to target pharmacologically, which sets a limit to their prospects as drug targets^18^.

Network topology is an area of network science describing the connection structure of a network consisting of nodes and edges^19^, which are in this case proteins and the interactions between them. This framework is widely applied in different biomedical research^20,21^, and is especially often used on cancer signalling networks to find proteins or modules responsible for important biological processes such as metastasis formation^22,23^. Topological studies showed that IDPs are key players in the information flow of the signalling networks, while they strongly interact with the most central nodes. However, the papers did not further characterise these interactions^17,24^. Network motifs (i.e. small subnetworks having a significantly higher abundance than that in random networks) are important hotspots in the regulation of signalling networks^25,26^. Participation in interconnected motifs often indicates a stronger regulatory relationship between two nodes, than just a simple interaction^27^. In this paper, as a hypothesis-generating framework, we explored the simplest network motifs, i.e. three-nodal triangles that contain both biomarkers and oncologic targets as network topological models of their frequent co-regulation. This allowed us to screen target-interacting proteins on a system level as potential predictive biomarkers, defined as a protein or gene whose expression level or mutational status can help predict sensitivity to a certain drug. Our working hypothesis was that protein disorder and protein position in signalling networks may contribute to the efficacy of the prediction of predictive oncological biomarkers.

To establish an efficient ranking of oncogenic target-neighbour pairs, we used the more interpretable decision tree–based machine learning models, such as Random Forest^28^ and XGBoost^29^, which were already successfully used on different biomedical data^30,31^. Training and classification were carried out by using topological information of the signalling networks and protein annotations, supplementing the mechanistic description of network signalling with real life biological data for the optimisation of our models’ decision-making. This included comprehensive data of three different IDP databases and prediction methods, namely DisProt^32^, AlphaFold^33^ and IUPred^34^. To sum up the power of the machine learning predictions in a single number, a Biomarker Probability Score (BPS) was established to help the ranking of potential biomarkers by our MarkerPredict method.

## Results

### Intrinsically disordered proteins are enriched in triangles

**Fig. 1:**
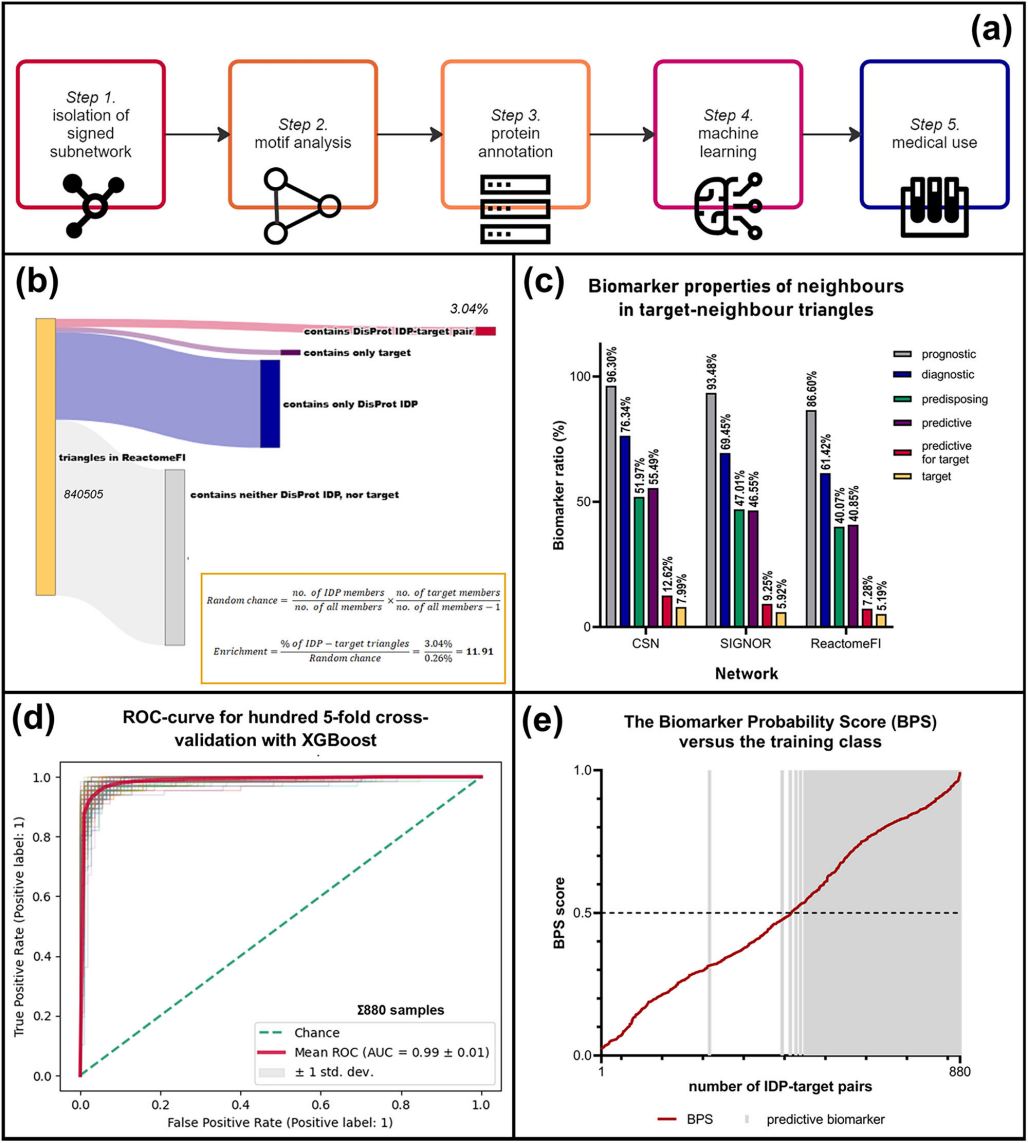
A step-by-step approach to the network-topology-based identification of predictive oncotherapeutical biomarkers. **a** A 5-step flowchart of the MarkerPredict process. In the first two steps, the network topology analysis process is detailed. After the identification of IDP-target pairs, the next step is protein annotation to establish the final input dataset. Then, machine learning models are trained, and the classification of the unlabelled data is carried out. As a final step, the predicted predictive biomarkers are reviewed with the scope of potential medical usage. **b** Step 1.-2.: Sankey-diagram of the identified triangles in the ReactomeFI^37^ network. 3.04% of the triangles contained DisProt-defined IDPs or targets. Based on the number of DisProt IDP and target members in triangles, the random chance for IDP-target triangles can be calculated. Comparing this value with the actual ratios, IDP-target triangles are overrepresented in every network. The DisProt enrichment ratio is 11.91 in ReactomeFI (highlighted), 5.66 in CSN^35^ and 4.86 in the SIGNOR^36^ network. For AlphaFold IDPs (defined as pLLDT < 50), it is 5.48, 6 and 1.7, for IUPred long score>0.5 it is 6.1, 5.88 and 3.74, and for short score > 0.5 it is 3.98, 7.41 and 3, respectively. **c** Step 3.: The biomarker properties of neighbours of targets in triangles including cancer drug targets. The majority of neighbours (86.6% to 96.3%) were biomarkers according to the CIViCmine database. Among predictive biomarker neighbours, a considerable ratio is established as a predictive biomarker of a drug which has its target in a shared triangle with the particular protein. **d** Step 4.: Receiver operation characteristic (ROC) curve of hundred 5-fold cross-validation with the XGBoost^29^ model trained on the combined data of CSN, SIGNOR and ReactomeFI networks, on the data of all 3 IDP databases and prediction methods. The model reached high performance, with the average area under curve (AUC) of 0.99 ± 0.01 (marked with red). Other validation models also showed high performance (see Supplementary Table S3). **e** Step 5.: The Biomarker Probability Score (BPS), a rank score calculated from prediction probabilities (for the definition see Fig. 2) versus the original label of the training dataset. This figure shows the BPS score calculated with the models trained on all 3 IDP databases and prediction methods. In the order of growing BPS values (marked with red), the original labels are showed with grey shadowing. The large correlation is visible, with a few differing labels around average BPS values.

As an initial step of our study, we identified the motif characteristics of those IDPs which were listed in the DisProt^32^ database as illustrated in Fig. 1a. Three signed subnetworks (i.e. network segments containing positive and negative links) with greatly differing network topological characteristics (see Supplementary Table S1) from the Human Cancer Signaling Network (CSN network)^35^, SIGNOR^36^ and ReactomeFI^37^ were used for the analysis. Three-nodal motifs were identified with the FANMOD programme, followed by the selection of triangles, i.e. fully connected three-nodal motifs for the analysis (see ‘Methods’ and Supplementary Table S1). Rare regulatory motifs, such as unbalanced triangles and cycles were also identified due to their special role in signalling networks.

After obtaining the list of the IDPs and oncotherapeutic targets (as detailed in Methods), they were identified in the triangles. Triangles containing both DisProt^32^ IDP and target members were separately analysed as special hot spots in signalling networks. Unbalanced triangles, i.e. triangles with odd number of negative links were significantly overrepresented among these, while cycles were only in the CSN and SIGNOR networks (see Supplementary Fig. S1). These IDP-target triangles exist with a much larger frequency than they would with a random chance (see Fig. 1b and Supplementary Fig. S1). Very importantly, this enrichment was also true withAlphaFold (average pLLDT<50) and IUPred (average score > 0.5) defined IDPs. Having a common motif is usually an indicator of a very close regulatory connection between two nodes in network science, and motif structures can even be used to predict new drug targets^27^. As an ideal predictive biomarker must show when conditions affecting the efficacy of the respective drug occur^38^, within ideal condition they must also affect or be affected by the change of signalling in the drug target’s pathway. One way to achieve that is to be in the same signalling pathway, which is the case with some famous biomarkers, such as BRAF for EGFR inhibitors in colon cancer^2^. In our method, instead of traditional depiction of signalling pathways, participating proteins are represented in a simplified manner in the form of three-nodal motifs. Thus, as three-nodal motifs usually represent a part of a signalling pathway, the hypothesis of these IDPs being predictive biomarkers for the drugs targeting their triangle neighbour (called as ‘IDP-target-pairs’ throughout the paper, where pairs were included regardless of the sign and direction of the connecting links) emerged. To broaden the scope of this study, all neighbours of the targets found in the networks were included in the analysis with their respective target pairs (called as ‘neighbour-target-pairs’ throughout the paper).

### Intrinsically disordered proteins are likely to be cancer biomarkers

To evaluate this hypothesis, the biomarker properties of the IDPs were annotated using the CIViCmine text-mining database (as described in Methods in detail). The database annotated separately prognostic, predisposing, diagnostic and predictive biomarkers. The results show that in all three networks, more than 86% of the IDPs were prognostic biomarkers, with high ratios of all the other biomarker types (see Fig. 1c). CIViCmine has biomarker data for 12 452 different proteins.

Cases when the disordered protein was the predictive biomarker for its target triangle pair were annotated and manually reviewed. These were handled as positive controls (class 1) in the training dataset for the machine learning model (see Section 3.5.). Altogether, in 332 cases of the 4550 neighbour-target pairs the neighbour member of the pair was an established predictive biomarker for the drug targeting its target pair. This is a convincing subset considering the specificity of this attribute. To evaluate those 4223 neighbour-target pairs where the neighbours were not predictive for their target in the CIViCmine database^39^, a negative control dataset was also established from the neighbour proteins not present in CIViCmine and random pairs (see ‘Methods’), and machine learning models were established for a binary classification.

### High-performing machine learning models are established for prediction

After constructing the training dataset, both Random Forest and XGBoost binary classification methods were trained on both the network-specific and the combined data of all 3 signalling networks, and on the individual and the combined data of all 3 IDP-databases and prediction methods resulting in thirty-two different high-performing models (see ‘Methods’ and Supplementary Table S4). The optimal hyperparameters were set with competitive random halving (see Supplementary Text S1). During the optimisation process, the predictive power of these models was thoroughly investigated.

#### High performance with validation methods

The strength of the models was evaluated with multiple validation methods. Leave-one-out-cross-validation (LOOCV), k-fold cross-validation and validation with the 70:30 splitting of the training and test dataset were used. All the models produced good metrics with the established hyperparameter settings, even those trained on the smaller DisProt database had an acceptable performance(see Fig. 1d and Supplementary Table S4).

The Random Forest algorithm marginally underperformed compared to XGBoost. The models performed less well on the CSN network, which could be explained by its smaller number of nodes and links. Overall, due to their high AUC, accuracy and F1-score with validation methods, all models were evaluated to be precise enough for the inclusion in the final classification of neighbour-target pairs. As the used IDP-database DisProt^32^, and prediction methods AlphaFold^33^ and IUPred^34^ were not correlating strongly (see Supplementary Fig. S2), four (three individual and one combined) predictions were conducted.

To harmonise the probability values of the final prediction, the normalised average of the ranked probability values was defined as the BPS. To estimate the discriminative strength of the Biomarker Predictability Score, during the LOOCV on the test dataset (for the combined prediction), the predicted probability value for each data point of the test dataset was exported to carry out BPS calculation with the same method described above. Comparing this probability value with the original class of the neighbour-target pair (see Fig. 1e), they correlate well, with altogether 88 wrong classifications out of the 880 originally classified neighbour-target pairs *(10%)* all having BPS values around the average.

#### The machine learning approach is widely applicable

To address the predictive power of different groups of input features, XGBoost and Random Forest LOOCV models were trained on each group of features. Both the group of biological and topological features reached the accuracy of 0.86 (see Supplementary Fig. S3). Models trained on different small groups of topological parameters, such as centrality data or motif parameters also reached high accuracy values. This shows that with the use of only network topological data as input effective biomarker identification is still possible.

The MarkerPredict method described in this paper may be used on other networks than the three signalling networks it was developed on. Systematic cross-training among the networks, i.e. training on one and testing on another one was implemented to model this scenario. Consistent high performance was achieved between the two largest networks: ReactomeFI and SIGNOR (see Supplementary Fig. S5), although the DisProt database’s model showed worse performance. Reducing the training dataset to topological parameters worsened the metrics in most of the cases. However, with larger, similarly structured networks and abundant data, this method may be useful to predict novel predictive biomarkers of unknown networks.

#### Disorder content as one of the most important input features

Feature importance analysis was conducted by using the SHAP package, which uses a game theory-based Shapley values to assess the importance of each feature. The SHAP plots show (see Supplementary Fig. S6) that the values of the IDP databases and prediction methods are important input features. The centrality values and the number of different type of triangles reached high values. For the abovementioned topological parameters, higher feature values meant higher SHAP values. In some cases, neighbour pairs with low or moderate disorder content are more likely to be predictive biomarkers, while for the IUPred short predictions, larger disorder content further increase this likelihood.

To summarise, IDPs are more likely to be biomarkers, and IDP disorder data is an important input feature of predictive biomarker prediction. However, the exact translation of this depends on the score or feature in question.

### Identified potential predictive biomarkers

**Fig. 2:**
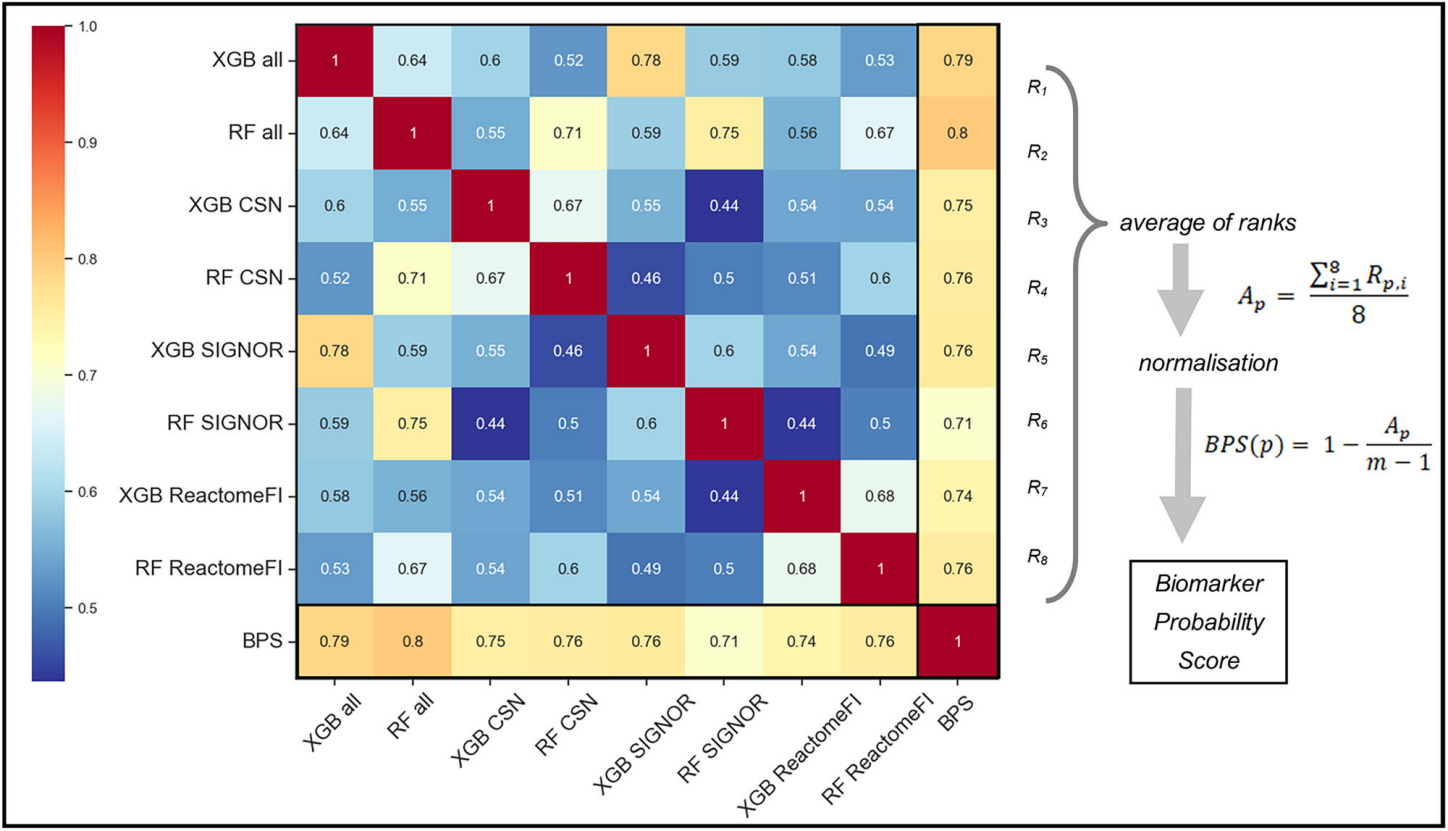
Correlation heatmap of classification probability values of eight different models and the Biomarker Probability Score (BPS) on the data of all 3 IDP annotations. Each of the XGBoost^29^ (XGB) and the Random Forest^28^ (RF) models were trained on the three individual network-specific data of CSN^35^, SIGNOR^36^, and ReactomeFI^37^, as well as on the combined data of all three networks. Probability values of classifying the particular target-neighbour as a predictive biomarker for the respective target (i.e. class 1, see main text for definition) were gained from the eight different machine learning models. BPS was calculated according to the two equations shown on the right side of the figure from the normalised average of the ranks of probabilities. Four separate BPS values were calculated using models trained on the data from DisProt^32^, AlphaFold^33^, IUPred^34^, and a combined dataset. The values shown in this figure correspond to the model trained on the combined dataset. Ranking of biomarker probability values was performed in ascending order. In case of identical probabilities, the average of the consecutive ranks was given. In the equations A_p_ is the average of the ranks for a given neighbour-target pair p, and m is the number of all predictions. Finally, the correlation matrix was created from the probability values and BPS, which is shown on the figure. As shown, BPS had strong unbiased correlation with each prediction within the range of 0.71–0.8.

After the final predictions with the eight different models, the Biomarker Predictability Score (BPS) was calculated for each of the neighbour-target pairs combined and for each IDP annotation (see Section 3.5–6.) The BPS score was able to bridge the difference among the probability calculation methods of the XGBoost and Random Forest algorithms and different networks (see Fig. 2). In 2084 neighbour-target pairs the neighbour could be classified as potential predictive biomarker for the respective target, if we consider a threshold of BPS score larger than 0.5 for at least one of the four calculation (see Supplementary Table S5). 426 pairs had a BPS above 0.5 for all calculations. For many of the pairs with top BPS values, preclinical or clinical literature was found supporting the machine learning prediction (see Table 1). The top 5 prediction for each BPS score calculation contained targets of mainly small molecule receptor or HER2 inhibitors, where the neighbour had a low to medium disorder content. Two of these predictions, LCK and ERK1 are discussed in detail in the further part of this section, with four additional examples, CREB, integrin β1, Notch1 and β-catenin which are lowlier ranked but interesting from a medical standpoint, are in the Supplementary Material (see Supplementary Figs. S6, S10). The fact, that even lower ranking (but well-above the threshold) hits are also good examples for biomarker candidates, shows the usefulness of our full list of 2084 potential biomarkers (Supplementary Table S5).

**Table 1:** Top 5 neighbour-target pairs with the highest Biomarker Probability Score (BPS) for 3 different IDP annotations and for the models of the combined annotations

No	Biomarker Probability Score (BPS), combined /annotation-specific	Neighbour name	Annotation disorder content (%)				Target name	Inhibitor(s) of target	Supporting literature
			***DisProt***	***AlphaFold***	***IUPred (long)***	***IUPred (short)***			
ALL ANNOTATIONS									
1.	0.998	**LCK**	7.1%	11%	3.1%	3.9%	**EGFR**	afatinib, cetuximab, erlotinib, gefitinib, lapatinib, necitumumab, osimertinib, panitumumab, vandetanib	Clinical: Pizzamiglio et al.^42^,
2.	0.991	**LCK**	7.1%	11%	3.1%	3.9%	**PDGFRB**	regorafenib, axitinib	
3.	0.990	**ITGB1**	5.9%	3.8%	5%	6.6%	**EGFR**	afatinib, cetuximab, erlotinib, gefitinib, lapatinib, necitumumab, osimertinib, panitumumab, vandetanib	Preclinical: Gu et al.^73^,
4.	0.990	**ERK1**	6.1%	6.5%	6.6%	12%	**FGFR3**	ponatinib	Preclinical: Matsuda et al.^74^,
5.	0.987	**STAT1**	5.5%	6.1%	6.8%	6.9%	**EGFR**	afatinib, cetuximab, erlotinib, gefitinib, lapatinib, necitumumab, osimertinib, panitumumab, vandetanib	Preclinical: Yang et al.^75^, Clinical: Srivastava et al., 2016^76^
DISPROT									
1.	0.991	**ERK1**	6.1%	6.5%	6.6%	12%	**BRAF**	dabrafenib, vemurafenib	Preclinical: King et al.^77^,
2.	0.988	**ERK1**	6.1%	6.5%	6.6%	12%	**RAF1**	regorafenib, sorafenib	Preclinical: Cao et al.^78^,
3.	0.987	**ERK1**	6.1%	6.5%	6.6%	12%	**HER2**	trastuzumab, pertuzumab, ado-trastuzumab emtansine, lapatinib, neratinib, afatinib	Preclinical: Li et al.^79^,
4.	0.984	**SRC**	16%	14%	16%	14%	**RAF1**	regorafenib, sorafenib	
5.	0.980	**ERK1**	6.1%	6.5%	6.6%	12%	**BCL-2**	venetoclax	
ALPHAFOLD									
1.	0.979	**TRAF6**	N/A	12%	4.4%	4.2%	**HER2**	trastuzumab, pertuzumab, ado-trastuzumab emtansine, lapatinib, neratinib, afatinib	
2.	0.976	**PRKCA**	N/A	8.6%	9.1%	11%	**HER2**	trastuzumab, pertuzumab, ado-trastuzumab emtansine, lapatinib, neratinib, afatinib	Preclinical: Pandya et al.^80^,
3.	0.976	**JNK**	N/A	14%	17%	19%	**HER2**	trastuzumab, pertuzumab, ado-trastuzumab emtansine, lapatinib, neratinib, afatinib	Preclinical: Itah et al., 2023^81^
4.	0.974	**NFkB**	N/A	26%	20%	15%	**MTOR**	everolimus, temsirolimus	Preclinical: Xie et al., 2007^82^
5.	0.973	**LCK**	7.1%	11%	3.1%	3.9%	**PDGFRB**	regorafenib, axitinib	
IUPRED									
1.	0.993	**FYN**	N/A	15%	7.3%	8.2%	**EGFR**	afatinib, cetuximab, erlotinib, gefitinib, lapatinib, necitumumab, osimertinib, panitumumab, vandetanib	Preclinical: Kim et al.^83^,
2.	0.990	**ERK1**	6.1%	6.5%	6.6%	12%	**HER2**	trastuzumab, pertuzumab, ado-trastuzumab emtansine, lapatinib, neratinib, afatinib	Preclinical: Li et al.^79^,
3.	0.987	**TRAF6**	N/A	12%	4.4%	4.2%	**EGFR**	afatinib, cetuximab, erlotinib, gefitinib, lapatinib, necitumumab, osimertinib, panitumumab, vandetanib	
4.	0.986	**ERK1**	6.1%	6.5%	6.6%	12%	**JAK2**	ruxolitinib	Preclinical: Stivala et al.^84^,
5.	0.983	**STAT1**	5.5%	6.1%	6.8%	6.9%	**EGFR**	afatinib, cetuximab, erlotinib, gefitinib, lapatinib, necitumumab, osimertinib, panitumumab, vandetanib	Preclinical: Yang et al.^75^, Clinical: Srivastava et al., 2016^76^

BPS was calculated from the 8 predictions (two machine learning algorithms on three signalling networks and their total) on the unlabelled dataset. Disorder content was identified from the DisProt^32^ database and the AlphaFold^33^ and IUPred^34^ prediction methods, while corresponding drugs were listed from the My Cancer Genome^67^ database. Supporting literature was identified in PubMed using the drug and IDP names as keywords. Articles explaining a rationale leading to predictive biomarker properties were selected, along with articles where the effectivity of the drug could be estimated from the mutation status or expression level of the IDP. When there was, one example article of preclinical, and one with clinical evidence was cited.

#### LCK as a potential predictive biomarker for EGFR inhibitors

**Fig. 3:**
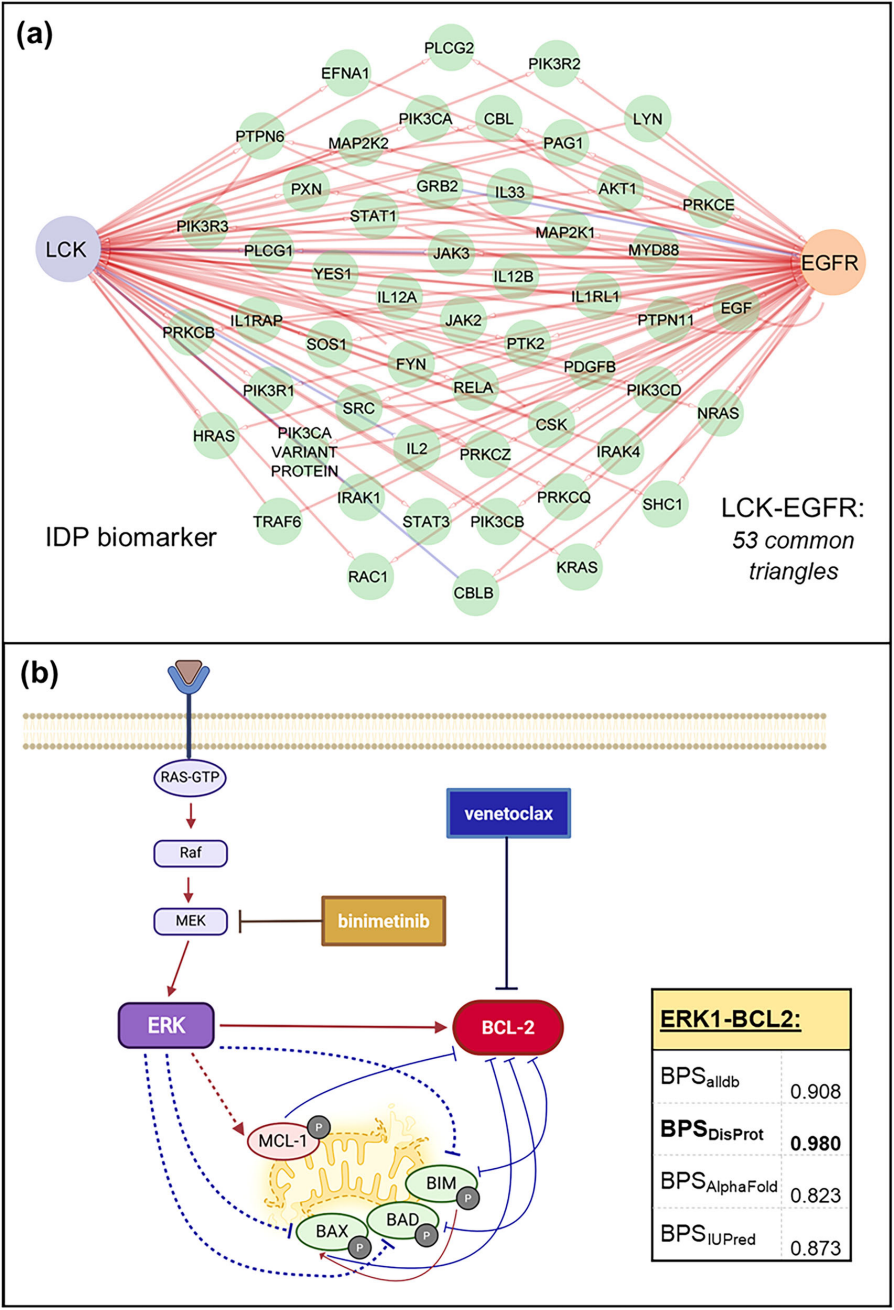
The detailed examples of top predicted biomarkers. Network motifs containing the LCK-EGFR IDP-target pair, and the common neighbours of LCK and EGFR in the ReactomeFI network. **a** The IDP target pair participated in 53 triangles. **b** ERK1 as a potential biomarker for the BCL-2 inhibitor venetoclax. ERK1, as a member of the MAPK pathway, inhibits the mitochondrial apoptotic process through the phosphorylation of various regulators. The stabilization of MCL-1 led to venetoclax resistance. The inhibition of MEK1/2 with binimetinib has synergistic effects with venetoclax in preclinical models. Positive interactions are highlighted with red, negative interactions with blue. The interactions in ReactomeFI are shown with a full, and the ones from the literature with a dashed line. The predicted four BPS values are highlighted in the right corner of the figure.

LCK was predicted by the MarkerPredict model to be a potential predictive biomarker for EGFR inhibitors, achieving the highest BPS with the models trained on all 3 IDP annotations. The BPS_alldb_ score of the LCK–EGFR pair was 0.998, and all available 24 models confidently classified LCK as a class 1 predictive biomarker. The BPS_AlphaFold_ was 0.969, and the BPS_IUPred_ was 0.979, and this pair was not in the DisProt database. In the ReactomeFI network, LCK stimulates EGFR, and they are involved in 53 shared triangle motifs. The motif subnetwork contains both direct regulatory edges and indirect feedback structures, as visualised in Fig. 3a. Both LCK and EGFR showed non-zero centrality values in all three networks. While LCK is not part of CIViCmine as a predictive biomarker for EGFR inhibitors, previous clinical studies have shown its correlation with treatment outcomes.

EGFR is a well-characterised oncogenic receptor tyrosine kinase in non-small cell lung cancer, colon cancer and several other malignancies. Numerous drugs target EGFR, including afatinib, cetuximab, erlotinib, gefitinib, lapatinib, necitumumab, osimertinib, panitumumab and vandetanib. EGFR activation triggers downstream signalling through the MAPK, PI3K-AKT, and JAK-STAT pathways. LCK is a Src family tyrosine kinase that is known for its role in T-cell signalling but is increasingly recognised for its function in epithelial cancers, including lung and breast cancers^40^. LCK is also known to interact with cytoskeletal and adhesion-associated proteins^41^. Clinical evidence indicates that LCK expression correlates with response to targeted therapies, including EGFR- and HER2 inhibitor lapatinib^42^. It is interacting with EGFR in cholangiocarcinoma, contributing to the synergistic effect of their dual inhibition^43^. LCK is a validated IDP, with a disorder content of 7% in DisProt^32^ and 11% based on AlphaFold^33^ predictions, showing that IDPs with low-moderate disorder content can be suitable predictive biomarkers.

#### ERK1 as a potential predictive biomarker for BCL-2 inhibitors

Among the novel predictions of the MarkerPredict framework, ERK1 (MAPK3) was identified as a potential predictive biomarker for BCL2 inhibitors, including venetoclax. The BPS_DisProt_ score of the ERK1–BCL-2 pair was 0.980, and it has high, >0.823 BPS scores with other predictions with other IDP annotations too - the pair was consistently predicted as class 1 in all 32 models. ERK1 and BCL-2 are connected via multiple shared neighbours, forming 44 triangular motifs in ReactomeFI, and 6 motifs in SIGNOR. Both ERK1 and BCL2 displayed non-zero centrality across the networks. While BCL-2 is a well-known drug target in haematological malignancies, ERK1 has not previously been described as a predictive biomarker for BCL-2 inhibition in any annotation.

BCL-2 is an anti-apoptotic protein involved in mitochondrial outer membrane integrity, and its inhibition by venetoclax serves as a therapeutic option in chronic lymphocytic leukaemia, acute myeloid leukaemia, and other diseases. Resistance to venetoclax often emerges through compensatory upregulation of other anti-apoptotic proteins, particularly MCL-1, which is stabilised by ERK1 activity^44^. ERK1, as part of the MAPK cascade, regulates transcription factors and phosphorylates pro-apoptotic BCL-2 family members such as BIM, BAD, and BAX^45^. In preclinical models, inhibition of other proteins as MAPK signalling such as MEK1/2 inhibitor binimetinib sensitised cells to venetoclax^46^, suggesting that ERK1 levels or activity also might influence response to BCL-2 inhibition. ERK1 has a moderate disorder content of 6.5–12% across the disorder predictors used, which corresponds with the disorder content range observed in many top-scoring predictions. Also, it fits the trend that higher IUPred short disorder content may raise the chance to have a higher BPS.

Although ERK1 is not currently recognised as a clinical biomarker for BCL-2-targeted therapy, its central position in apoptosis regulation and role in adaptive resistance mechanisms make it a promising candidate. Further investigation into ERK1 expression or activity status in venetoclax-treated patients may clarify its potential utility as a predictive biomarker.

## Discussion

With the emergence of advanced therapeutics in oncology, good predictive biomarkers became essential for therapeutic decision-making^4^. In this work, we propose that protein disorder and protein position in signalling networks may contribute to predictive biomarker prediction due to IDPs importance in cancer signalling and key position in signalling networks, namely, their closeness to the targets of oncotherapeutical drugs^24^. As we showed, IDPs are overrepresented as target-neighbours, and most of these neighbours in our networks are already established prognostic biomarkers (Fig. 1c). These disordered regions also help proteins to be highly adaptable to the changing environment, allowing phenotype switching as a method of adaptation in cancer^16^. Disordered driver genes often have mutations in their disordered regions^17^. All of this suggests that the mutation or the change of expression of disordered proteins may highlight important phenotypic changes in cancer cells, which may also include therapy resistance.

As disordered regions contribute substantially to the emergence of the attributes of good predictive biomarkers, it was one of the goals of this work to determine the optimal disorder content ratio for being a good candidate. The SHAP analysis of our machine models highlighted disorder content as one of the most important input features, regardless of the annotation used. The ‘ideal’ IDP disorder content is not clear and very annotation- and model-dependent, however, our data shows that IDPs with small disorder content can also be good predictive biomarkers, such many of our top biomarker predictions (see Table 1), which may be explained by some earlier observations. Even IDPs become more ordered upon binding to their partners, however, they retain a certain, moderate level of disorder, called ‘fuzzyness’^47^. Fuzzyness allows the rewiring of signalling networks and the development of functional switches^48^, which are key features of cancer accommodation. On the other hand, proteins having ordered domains with established tertiary structures may participate in signalling as traditional signalling proteins, while their disordered regions allow them to make novel connections and to quickly adapt to the frequent changes of the environment in cancer and its treatment. This duality, combined with participation in close co-regulatory motifs with drug targets may explain the models’ top predictions with low-moderate disorder content. However, further studies are needed to explain the molecular and structural background of this phenomenon.

With the MarkerPredict method, we established a high-accuracy binary classification algorithm for the prediction of new predictive biomarker–therapy target pairs based on network topological and protein annotation data. This approach is novel. However, many algorithms were developed to classify and analyse biomedical data^49^. Here we show a few examples of the Random Forest and XGBoost algorithms used in cancer research and biomarker identification.

Random Forest is a widely spread algorithm for classification problems in biomedical sciences. Translocatome, a high-performing random forest classifier was used on network data to predict protein translocation^30^. RepCOOL, a network-based drug reposition algorithm by using random forest reached the AUC of 0.83^50^. In another study, different random forest methods’ performance was compared, while using omics data for biomarker selection^51^. Random Forest showed higher performance than XGBoost in a study to identify lung cancer- specific biomarkers^52^.

The Random Forest algorithm was also used in clinical classificatory problems. Weighted correlation network analysis was used to build a random forest model to predict anti-PD1-therapy response in melanoma^53^, reaching an AUC of 0.71 on the validation set. Random forest was also used to predict CDK4/6 inhibitor and endocrine therapy efficacy based on bacterial species in faecal microbiome of metastatic breast cancer patients^54^, and to characterise endocrine therapy resistance to identify pre-adapted breast cancer cells based on differently expressed biomarkers^55^. It was shown to be capable to identify predictive biomarkers of radiotherapy sensitivity in rectal cancer^56^.

XGBoost was used somewhat less frequently on both biological and clinical data. Multiple different machine learning algorithms were used to predict anti-PD1-therapy response in non-small cell lung cancer, where the XGBoost model reached the AUC of 0.824 in cross-validation^57^. XGBoost was the highest performing model when used in the validation of hub genes in gastric cancer and healthy gastric tissue, reaching an accuracy of 89% on the test set^58^. In comparison, despite the small training dataset of 880 annotated neighbour-target pairs, achieving high AUC values with a combined 96% accuracy with LOOCV with both Random Forest and XGBoost algorithms is noteworthy, outperforming many of the abovementioned models.

We believe that the good AUC metrics we received may be a result of the use of protein pairs for the prediction instead of classifying single proteins. This increases data variety and gives the decision tree-based model more possibilities to construct the optimal decision tree. Previous studies also used pairwise analysis approaches with good but less outstanding results; however, they usually did not limit the search on previously established network neighbours^59,60^. In principle, the high AUC values we obtained may show overfitting, as it is a common issue in machine learning, especially in models trained on small sample sizes^61^. Thus, we implemented rigorous cross-testing methods such as *LOOCV* and cross-training among the network to prove the strength of our models in various conditions. Our data show that our model highly performs not only the training dataset, but also on previously unseen data, which is in contrast with the behaviour of overfitted models^61^. As a next step to avoid overfitting, we created thirty-two models using two classification algorithms, three IDP-databases and prediction methods and three networks, followed by homogenising their predictions into four different BPS to minimise the importance of a potential overfitting of one model on network-specific data. Thus, while overfitting remains a significant consideration, this study has implemented precautionary measures to mitigate this concern, affirming the validity of our performance results.

A critical concern was whether the use of the manually curated, but sparse DisProt alongside the AlphaFold and IUPred prediction methods might bias the results due to different underlying methodologies, especially as the methods do not correlate perfectly with each other (see Supplementary Table S2). To address this, we compared the predictions across all three resources. As shown in Table 1, the top-ranked DisProt-based predictions exhibited strong agreement with those derived from AlphaFold and IUPred, with BPSs consistently above 0.8 across methods. For example, the top DisProt-derived pairs (ERK1–BRAF, ERK1–RAF1, ERK1–ERBB2, SRC–RAF1, ERK1–BCL2) were also classified with high confidence by the other methods, supporting their robustness as predictive biomarker candidates. This high level of consistency justifies our decision to use both curated and predicted date of protein disorder. Consequently, excluding DisProt from the study would lead to a loss of relevant information; while including it strengthens the hypothesis-generating framework by integrating both curated and predictive disorder evidence. Taken together, these results confirm that the integration of multiple disorder annotation methods is justified and enhances the robustness of MarkerPredict’s biomarker prediction.

Two examples illustrate how the MarkerPredict framework identifies both established and novel predictive biomarker candidates. The first is LCK, a Src-family kinase predicted as a biomarker for EGFR inhibitors. LCK is involved in cytoskeletal organisation and integrin signalling and has been shown to support compensatory EGFR signalling in cholangiocarcinoma^43^ Its predictive role has also been supported in clinical cohorts of lapatinib-treated breast cancer patients^42^, further validating the model’s performance. The second example is ERK1, a novel prediction as a biomarker for BCL2 inhibitors such as venetoclax. While not currently used as a clinical biomarker, ERK1 regulates multiple pro-apoptotic BCL2 family members and mediates resistance to BCL2 inhibition via stabilization of MCL1^44,45^. Inhibition of ERK1 or upstream MAPK signalling has been shown to restore sensitivity to venetoclax in preclinical models, supporting the biological plausibility of the prediction^46^. These examples highlight how the model can recover known relationships and prioritise mechanistically supported but underexplored candidates for future biomarker development.

In summary, our MarkerPredict biomarker classification algorithm, accessible on GitHub through the MarkerPredict package and input data (https://github.com/klari98/MarkerPredict, see Supplementary Text S2, Table S2 and S5), offers a platform for extensive exploration. Using this tool here we classified 3670 neighbour-target pairs with a 0.78-0.96 LOOCV accuracy. We identified 2084 proteins as potential predictive biomarkers to targeted cancer therapeutics, while 426 pairs had a BPS above 0.5 for all four calculations. We detailed the biomarker potential of CREB, integrin β1, Notch1 and β-catenin. Our method provides a new rationale—search for signalling network neighbours of known drug targets—to identify novel biomarkers. This approach can also be expanded to novel signalling networks, unlocking the potential to discover additional relevant biomarkers. These biomarker predictions are not validated yet, however, after forward translation such as validation on cell lines, animal models or reverse translation of looking at patient data, they have a promising prospect to be used in clinical settings as well. With this work, our goal is to select the most promising candidates that can go through the validation process, making biomarker detection more effective. Finding better predictive biomarkers for targeted cancer therapeutics may contribute to the optimisation of therapeutic decision-making, thus helps avoiding unnecessary side effects and improving the prognosis of cancer patients within the realms of personalised medicine. We invite researchers to utilize our findings in advancing personalised patient care.

## Methods

### Isolation of signed subnetworks

To conduct an analysis of the network topological properties of IDPs, three different signalling network datasets were acquired. The Human Cancer Signaling Network^35^, SIGNOR^36^ and ReactomeFI^37^ networks all consist of curated signalling regulations between proteins. Discrimination between positive and negative links was necessary for the subsequent motif analysis. Thus, the signed subnetwork, i.e. the part of the network consisting of positive and negative edges of each network was isolated.

Standard network topological parameters such as the number of nodes and edges were calculated to compare the three different networks (see Supplementary Table S1). Network density was calculated for each network with the common formula^62^:

1 $$\eta =\frac{2{|E|}}{{|V|}({|V|}-1)}$$

where *η* is network density, *|V|* is the number of vertices i.e. nodes, and *|E|* is the number of edges in the graph. The number of triangles were determined with the FANMOD^63^ programme (see Section 3.2.). Overlapping nodes among the networks were identified by matching the node names with UniProtIDs through the UniProt^64^ database.

### Motif analysis

The FANMOD command-line programme was used to identify three-nodal directed network motifs. During the analysis, edge colours were used to distinguish positive and negative edges. Among the identified motifs, fully connected three-nodal motifs, also known as triangles, were selected for further analysis. The analysis also distinguished rare regulatory network motifs^65^. These motifs were identified as unbalanced triangles containing an odd number of negative edges, as well as cycles that allowed for a walk around the motif from at least one direction.

Once the identification of IDPs and oncotherapeutic targets had been carried out (see Section 3.3), the next step was the calculation of the enrichment of IDP-target pairs in triangles. Specifically, triangles with at least one IDP and one target member (irrespectively of the sign of their interaction) were separated and their IDP target-pairs are referred as ‘IDP target-pairs’ in throughout the paper. These triangles were subjected to further analysis, considering the directionality and sign of the edges between the IDP and target. To determine the ratio of regulatory motifs in these triangles, chi-square tests were employed to assess their statistical significance. Finally, the networks and motifs were visualised using the Cytoscape^66^ programme. To broaden the prospect of this study, all targets and their neighbours in motifs were included in the further analyses, referred as ‘neighbour-target triangles’ later.

### Protein annotation and the construction of the final input dataset

Database annotations were utilized to create the final input database for our machine learning models. Identification of these proteins within the networks was conducted through UniProt ID matching. Firstly, the DisProt^32^ database was used to identify IDPs, which contained validated human IDPs. The AlphaFold^33^ structural database and disorder prediction method was also used, extracting pLLDT (B-factor) values from downloadable .cif files. The average pLLDT score and the disorder content (defined as the ratio of regions under the pLLDT score of 50) was calculated. The IUPred^34^ Python package was also used to predict disordered regions on the FASTA files accessed through the UniProt^64^ database. Short, long, and globular scores were predicted, but globular scores were later excluded due to high similarity to long scores. Average scores and disorder content values (defined as the ratio of regions over the score of 50) were calculated. The disorder content values and scores were then exported as a biological attribute of the proteins and included in the final input dataset.

The list of oncotherapeutic targets for motif analysis was established using the My Cancer Genome^67^ database. Target proteins of approved targeted therapies in oncology and haematology were considered, along with immunological drugs with existing anticancer clinical trials. In the case of multikinase inhibitors, each target was individually analysed. A preclinical target list was obtained from the Target Central Resource Database (TCRD/PHAROS)^68^, which was filtered to exclude drugs in clinical trials based on the TCRD and ChEMBL^69^ databases, given the greater clinical demand and existing literature supporting predictive biomarkers for approved drugs.

Triangles that included at least one separate approved target member were identified as *neighbour-target triangles* (see Section 3.2.). To investigate the biomarker properties of IDPs in these triangles, we used the CIViCmine^39^ text-mining database, which discriminates among prognostic, predisposing, diagnostic, and predictive biomarkers. CIViCmine defined ‘predictive biomarker’ as a protein whose expression level or mutational status can help predict a sensitivity or response to a certain drug. In cases where the IDP was a predictive biomarker for the drug targeting the target, we conducted a manual review of the drugs and targets listed by CIViCmine. These cases were considered as positive control, i.e. class 1 in the final input dataset.

The final input dataset was created from the 4550 neighbour-target pairs identified in total in the three networks. For these pairs, pairwise and individual biological and network topological data were collected for each network (see Supplementary Table S3). In the case of biological data, regarding the IDPs and the targets, the disorder content, oncologic target status (yes (1) or no (0), see above), and the type of drug considered (small molecule or antibody) were collected. For topological data, the following parameters were extracted for all three networks: participation in the networks, the number of triangles and regulatory triangles, the direction and sign of the neighbour-target edges, the properties of the third node in the triangle, and centrality measures (bridgeness and betweenness centrality). Centrality measures were calculated with the ModuLand package of Cytoscape^70^.

The training set consisted of positive and negative control neighbour-target pairs. The pairs which where IDPs were established by using the CIViCmine database as predictive biomarkers for a drug targeting their pair, were classified as positive control, i.e. class 1 for the training dataset. For establishing a negative control set, we defined neighbours not present in CIViCmine as any type (prognostic, diagnostic, predisposing or predictive) biomarker, which was supplemented with randomly selected triangles for the smaller DisProt dataset. A completely randomised approach was also tested but resulted in worse metrics (see Supplementary Fig. S3). The established list of positive and negative control was used as the training dataset, while the remaining pairs were classified with the established machine learning models to predict new potential predictive biomarkers (see Supplementary Tables S2a and S2b, respectively).

### The setup of the machine learning model

To predict new potential predictive biomarkers, three different binary classification methods were selected. The Support Vector Machine, Random Forest and XGBoost were trained by using the Scikit-learn and XGBoost packages^28,29^. Random Forest and XGBoost models were used with the optimal hyperparameters acquired through competitive halving random search hyperparameter selection (see Supplementary Text S1), and with the fixation of the random state. The training dataset consisted of positive and negative control neighbour-target pairs. Network- and IDP annotation-specific analyses were also performed using condition-specific data subsets for these evaluations.

To evaluate the performance, multiple cross-validation methods were applied. These were cross-validation with 70:30 split of the input data, k-fold cross-validation and LOOCV. The metrics receiver operation characteristic area under curve (ROC AUC), precision, recall, their harmonic mean (F1-score) and accuracy were calculated. Due to its underperformance, we excluded the SVM model from further analysis, as the decision tree-based methods outperformed it. LOOCV models were also separately trained on multiple groups of input factors to determine the predicting power of different groups of biological and topological data. Systematic cross-classification among networks was also implemented by using the data of one network as a training dataset with the test classification on another one, with the aim to see the pan-network applicability of this method.

Final predictions were made on the dataset consisting of neighbour-target pairs that were not part of the training set. In addition to the ones trained on the data combined of all networks, network-specific separate models were trained by only considering the data belonging to the given network. In the same way, combined and IDP annotation-specific predictions were also conducted for the three IDP-databases and prediction methods. The predicted class and the probability of each class were determined. The most important features in both models were identified with the XGBoost feature importance and the SHAP^71^ Python package. The latter uses the Shapley values of game theory to address feature importance. For the SHAP analysis, the booster type was changed to *gbtree*, to accommodate to the requirements of the package.

To provide the availability of our method for further research, the MarkerPredict Python package containing the data and the code for the machine learning predictions were made available through GitHub with the appropriate documentation, using the *poetry* package^72^ (https://github.com/klari98/MarkerPredict). To perform network topology-based biomarker prediction on completely new networks, it is necessary to prepare the relevant biological and topological data according to the methods described above.

### The Biomarker Probability Score (BPS)

Here we define the BPS as a ranking-based machine learning probability score to assess the biomarker potential of neighbour-target pairs. It was calculated by using the acquired probabilities of label 1 (the given pair is classified as a potential predictive biomarker) from the final prediction. Using the Random Forest and XGBoost methods on the combined and the three network-specific data, altogether 32 separate predictions were made after training the models on the final input dataset. For the combined and each IDP-annotation-specific predictions, 4 separate BPS scores were calculated. The probability values were arranged in a descending order and were given ranks. In the case of identical probability values, the average of the subsequent ranks was given to all the repetitions. The BPS of each neighbour-target pair was defined as the normalised average of the rank of the predictions:

2 $${BPS}(p)=1-\frac{\left(\frac{{\sum }_{i=1}^{n}{R}_{p,i}}{n}\right)}{m-1}$$

where *R* is the rank of the probability of class 1 for the neighbour-target pair in the given prediction, *i*, *n* is the total number of predictions (8), and *m* is the number of the neighbour-target pairs where predictions were made. While BPS of 1 means the highest biomarker probability with all 8 predictions, BPS of 0 means lowest biomarker probabilities in all cases. BPS correlates well with the original training label (see Fig. 1e), thus was suitable for measuring the strength of future predictions.

## Supplementary information

Supplementary Information

Supplementary Data

Supplementary Data

## Data Availability

Data and codes for the learning prediction are available at this GitHub page: https://github.com/klari98/MarkerPredict.

## References

[1] Nussinov, R., Jang, H., Tsai, C. J. & Cheng, F. Review: Precision medicine and driver mutations: computational methods, functional assays and conformational principles for interpreting cancer drivers. *PLoS Comput. Biol.* **15**, e1006658 (2019). 30921324 10.1371/journal.pcbi.1006658 PMC6438456

[2] Alfaro Alfaro, A. E., Murillo Castillo, B., Cordero Garcia, E., Tascon, J. & Morales, A. I. Colon cancer pharmacogenetics: a narrative review. *Pharmacy* **10**, 95 (2022). 36005935 10.3390/pharmacy10040095 PMC9413567

[3] Slade, D. PARP and PARG inhibitors in cancer treatment. *Genes Dev.* **34**, 360–394 (2020). 32029455 10.1101/gad.334516.119 PMC7050487

[4] Vasan, N., Baselga, J. & Hyman, D. M. A view on drug resistance in cancer. *Nature* **575**, 299–309 (2019). 31723286 10.1038/s41586-019-1730-1 PMC8008476

[5] Shim, K. H., Kang, M. J., Youn, Y. C., An, S. S. A. & Kim, S. Alpha-synuclein: a pathological factor with Abeta and tau and biomarker in Alzheimer’s disease. *Alzheimers Res. Ther.* **14**, 201 (2022). 36587215 10.1186/s13195-022-01150-0 PMC9805257

[6] Ayyadevara, S., Ganne, A., Balasubramaniam, M. & Shmookler Reis, R. J. Intrinsically disordered proteins identified in the aggregate proteome serve as biomarkers of neurodegeneration. *Metab. Brain Dis.* **37**, 147–152 (2022). 34347206 10.1007/s11011-021-00791-8 PMC8748380

[7] Caminati, G. & Procacci, P. Mounting evidence of FKBP12 implication in neurodegeneration. *Neural Regen. Res.* **15**, 2195–2202 (2020). 32594030 10.4103/1673-5374.284980 PMC7749462

[8] Mahmud, Z. et al. Structure and proteolytic susceptibility of the inhibitory C-terminal tail of cardiac troponin I. *Biochim. Biophys. Acta Gen. Subj.* **1863**, 661–671 (2019). 30659884 10.1016/j.bbagen.2019.01.008

[9] La Penna, G. & Chelli, R. Structural insights into the osteopontin-aptamer complex by molecular dynamics simulations. *Front. Chem.* **6**, 2 (2018). 29441346 10.3389/fchem.2018.00002 PMC5797602

[10] Gursky, O. Structural basis for vital function and malfunction of Serum Amyloid A: an acute-phase protein that wears hydrophobicity on its sleeve. *Curr. Atheroscler. Rep.* **22**, 69 (2020). 32968930 10.1007/s11883-020-00888-y PMC7511256

[11] Yu, M. et al. Interferon-gamma induces tumor resistance to anti-PD-1 immunotherapy by promoting YAP phase separation. *Mol. Cell* **81**, 1216–1230 e1219 (2021). 33606996 10.1016/j.molcel.2021.01.010

[12] Alavi, S., Ghadiri, H., Dabirmanesh, B. & Khajeh, K. SPR analysis of SUMO-Murine Rap1-Interacting Factor 1 C-terminal domain interaction with G4. *Biosensors* **12**, 37 (2022). 35049665 10.3390/bios12010037 PMC8774283

[13] Russell, B. L. & Ntwasa, M. Expression, purification, and characterisation of the p53 binding domain of Retinoblastoma binding protein 6 (RBBP6). *PLoS ONE* **18**, e0277478 (2023). 36763571 10.1371/journal.pone.0277478 PMC9916574

[14] Kim, J. J. et al. CETN1 is a cancer testis antigen with expression in prostate and pancreatic cancers. *Biomark. Res.* **1**, 22 (2013). 24252580 10.1186/2050-7771-1-22 PMC4177615

[15] Kulkarni, P. & Uversky, V. N. Cancer/testis antigens: “smart” biomarkers for diagnosis and prognosis of prostate and other cancers. *Int. J. Mol. Sci.* **18**, 740 (2017). 28362316 10.3390/ijms18040740 PMC5412325

[16] Kulkarni, V. & Kulkarni, P. Intrinsically disordered proteins and phenotypic switching: implications in cancer. *Prog. Mol. Biol. Transl. Sci.* **166**, 63–84 (2019). 31521237 10.1016/bs.pmbts.2019.03.013

[17] Meszaros, B., Hajdu-Soltesz, B., Zeke, A. & Dosztanyi, Z. Mutations of intrinsically disordered protein regions can drive cancer but lack therapeutic strategies. *Biomolecules* **11**, 381 (2021). 33806614 10.3390/biom11030381 PMC8000335

[18] Santofimia-Castano, P. et al. Targeting intrinsically disordered proteins involved in cancer. *Cell Mol. Life Sci.* **77**, 1695–1707 (2020). 31667555 10.1007/s00018-019-03347-3 PMC7190594

[19] Galan-Vasquez, E. & Perez-Rueda, E. A landscape for drug-target interactions based on network analysis. *PLoS ONE* **16**, e0247018 (2021). 33730052 10.1371/journal.pone.0247018 PMC7968663

[20] Reckziegel, D. et al. Deconstructing biomarkers for chronic pain: context- and hypothesis-dependent biomarker types in relation to chronic pain. *Pain* **160**(Suppl 1), S37–S48 (2019). 31008848 10.1097/j.pain.0000000000001529 PMC6478400

[21] Katoh, M. Cardio-miRNAs and onco-miRNAs: circulating miRNA-based diagnostics for non-cancerous and cancerous diseases. *Front. Cell Dev. Biol.* **2**, 61 (2014). 25364765 10.3389/fcell.2014.00061 PMC4207049

[22] Li, J. et al. Identification of high-quality cancer prognostic markers and metastasis network modules. *Nat. Commun.* **1**, 34 (2010). 20975711 10.1038/ncomms1033 PMC2972666

[23] Yang, B. et al. Dynamic network biomarker indicates pulmonary metastasis at the tipping point of hepatocellular carcinoma. *Nat. Commun.* **9**, 678 (2018). 29445139 10.1038/s41467-018-03024-2 PMC5813207

[24] Rangarajan, N., Kulkarni, P. & Hannenhalli, S. Evolutionarily conserved network properties of intrinsically disordered proteins. *PLoS ONE* **10**, e0126729 (2015). 25974317 10.1371/journal.pone.0126729 PMC4431869

[25] Alon, U. Network motifs: theory and experimental approaches. *Nat. Rev. Genet.* **8**, 450–461 (2007). 17510665 10.1038/nrg2102

[26] Cloutier, M. & Wang, E. Dynamic modeling and analysis of cancer cellular network motifs. *Integr. Biol.* **3**, 724–732 (2011). 10.1039/c0ib00145g 21674097

[27] Zhang, X. D., Song, J., Bork, P. & Zhao, X. M. The exploration of network motifs as potential drug targets from post-translational regulatory networks. *Sci. Rep.* **6**, 20558 (2016). 26853265 10.1038/srep20558 PMC4744934

[28] Pedregosa, F. et al. Scikit-learn: machine learning in Python. *J. Mach. Learn. Res.* **12**, 2825–2830 (2011).

[29] Chen, T. & Guestrin, C. XGBoost: a scalable tree boosting system. In *Proc. 22nd ACM SIGKDD International Conference on Knowledge Discovery and Data Mining* 785–794 (Association for Computing Machinery, 2016).

[30] Mendik, P. et al. Translocatome: a novel resource for the analysis of protein translocation between cellular organelles. *Nucleic Acids Res* **47**, D495–D505 (2019). 30380112 10.1093/nar/gky1044 PMC6324082

[31] Toth, R. et al. Random forest-based modelling to detect biomarkers for prostate cancer progression. *Clin. Epigenetics* **11**, 148 (2019). 31640781 10.1186/s13148-019-0736-8 PMC6805338

[32] Quaglia, F. et al. DisProt in 2022: improved quality and accessibility of protein intrinsic disorder annotation. *Nucleic Acids Res.* **50**, D480–D487 (2022). 34850135 10.1093/nar/gkab1082 PMC8728214

[33] Varadi, M. et al. AlphaFold Protein Structure Database: massively expanding the structural coverage of protein-sequence space with high-accuracy models. *Nucleic Acids Res.* **50**, D439–D444 (2022). 34791371 10.1093/nar/gkab1061 PMC8728224

[34] Erdos, G., Pajkos, M. & Dosztanyi, Z. IUPred3: prediction of protein disorder enhanced with unambiguous experimental annotation and visualization of evolutionary conservation. *Nucleic Acids Res.* **49**, W297–W303 (2021). 34048569 10.1093/nar/gkab408 PMC8262696

[35] Cui, Q. et al. A map of human cancer signaling. *Mol. Syst. Biol.* **3**, 152 (2007). 18091723 10.1038/msb4100200 PMC2174632

[36] Lo Surdo, P. et al. SIGNOR 3.0, the SIGnaling network open resource 3.0: 2022 update. *Nucleic Acids Res.* **51**, D631–D637 (2023). 36243968 10.1093/nar/gkac883 PMC9825604

[37] Wu, G., Dawson, E., Duong, A., Haw, R. & Stein, L. ReactomeFIViz: a cytoscape app for pathway and network-based data analysis. *F1000Res* **3**, 146 (2014). 25309732 10.12688/f1000research.4431.1 PMC4184317

[38] Aronson, J. K. & Ferner, R. E. Biomarkers: a. general review. *Curr. Protoc. Pharmacol.* **76**, 9.23.1–9.23.17 (2017). 10.1002/cpph.19 28306150

[39] Lever, J. et al. Text-mining clinically relevant cancer biomarkers for curation into the CIViC database. *Genome Med.* **11**, 78 (2019). 31796060 10.1186/s13073-019-0686-y PMC6891984

[40] Weisse, J. et al. Identification of lymphocyte cell-specific protein-tyrosine kinase (LCK) as a driver for invasion and migration of oral cancer by tumor heterogeneity exploitation. *Mol. Cancer* **20**, 88 (2021). 34116687 10.1186/s12943-021-01384-w PMC8194179

[41] Mastrogiovanni, M., Juzans, M., Alcover, A. & Di Bartolo, V. Coordinating cytoskeleton and molecular traffic in T cell migration, activation, and effector functions. *Front. Cell Dev. Biol.* **8**, 591348 (2020). 33195256 10.3389/fcell.2020.591348 PMC7609836

[42] Pizzamiglio, S. et al. Integrated molecular and immune phenotype of HER2-positive breast cancer and response to neoadjuvant therapy: a NeoALTTO exploratory analysis. *Clin. Cancer Res.* **27**, 6307–6313 (2021). 34548320 10.1158/1078-0432.CCR-21-1600

[43] Jennifer, L. et al. In *Proc. ASCO Gastrointestinal Cancers Symposium* Vol. 41 (ASCO, 2023).

[44] Zhang, Q. et al. Activation of RAS/MAPK pathway confers MCL-1 mediated acquired resistance to BCL-2 inhibitor venetoclax in acute myeloid leukemia. *Signal Transduct. Target. Ther.* **7**, 51 (2022). 35185150 10.1038/s41392-021-00870-3 PMC8858957

[45] Thus, Y. J., Eldering, E., Kater, A. P. & Spaargaren, M. Tipping the balance: toward rational combination therapies to overcome venetoclax resistance in mantle cell lymphoma. *Leukemia* **36**, 2165–2176 (2022). 35725771 10.1038/s41375-022-01627-9 PMC9418002

[46] Kapoor, I., Bodo, J., Hill, B. T., Hsi, E. D. & Almasan, A. Targeting BCL-2 in B-cell malignancies and overcoming therapeutic resistance. *Cell Death Dis.* **11**, 941 (2020). 33139702 10.1038/s41419-020-03144-y PMC7608616

[47] Fuxreiter, M. & Tompa, P. Fuzzy complexes: a more stochastic view of protein function. *Adv. Exp. Med. Biol.* **725**, 1–14 (2012). 22399315 10.1007/978-1-4614-0659-4_1

[48] Miskei, M. et al. Fuzziness enables context dependence of protein interactions. *FEBS Lett.* **591**, 2682–2695 (2017). 28762260 10.1002/1873-3468.12762

[49] Cohn-Alperovich, D., Rabner, A., Kifer, I., Mandel-Gutfreund, Y. & Yakhini, Z. Mutual enrichment in aggregated ranked lists with applications to gene expression regulation. *Bioinformatics* **32**, i464–i472 (2016). 27587663 10.1093/bioinformatics/btw435

[50] Fahimian, G., Zahiri, J., Arab, S. S. & Sajedi, R. H. RepCOOL: computational drug repositioning via integrating heterogeneous biological networks. *J. Transl. Med.* **18**, 375 (2020). 33008415 10.1186/s12967-020-02541-3 PMC7532104

[51] Acharjee, A., Larkman, J., Xu, Y., Cardoso, V. R. & Gkoutos, G. V. A random forest based biomarker discovery and power analysis framework for diagnostics research. *BMC Med Genom.* **13**, 178 (2020). 10.1186/s12920-020-00826-6 PMC7685541 33228632

[52] Kashyap, A. H. et al. Novel biomarker prediction for lung cancer using random forest classifiers. *Cancer Inform.* **22**, 11769351231167992 (2023). 10.1177/11769351231167992 PMC10126698 37113644

[53] Wang, X. et al. Identification of potential biomarkers for anti-PD-1 therapy in melanoma by weighted correlation network analysis. *Genes* **11**, 435 (2020). 32316408 10.3390/genes11040435 PMC7230292

[54] Schettini, F. et al. Faecal microbiota composition is related to response to CDK4/6-inhibitors in metastatic breast cancer: a prospective cross-sectional exploratory study. *Eur. J. Cancer* **191**, 112948 (2023). 37454444 10.1016/j.ejca.2023.112948

[55] Hong, S. P. et al. Single-cell transcriptomics reveals multi-step adaptations to endocrine therapy. *Nat. Commun.* **10**, 3840 (2019). 31477698 10.1038/s41467-019-11721-9 PMC6718416

[56] Zhao, P., Zhen, H., Zhao, H., Huang, Y. & Cao, B. Identification of hub genes and potential molecular mechanisms related to radiotherapy sensitivity in rectal cancer based on multiple datasets. *J. Transl. Med.* **21**, 176 (2023). 36879254 10.1186/s12967-023-04029-2 PMC9987056

[57] Ahn, B. C. et al. Clinical decision support algorithm based on machine learning to assess the clinical response to anti-programmed death-1 therapy in patients with non-small-cell lung cancer. *Eur. J. Cancer* **153**, 179–189 (2021). 34182269 10.1016/j.ejca.2021.05.019

[58] Liu, J. & Cheng, Z. Identification of hub genes associated with tumor-infiltrating immune cells and ECM dynamics as the potential therapeutic targets in gastric cancer through an integrated bioinformatic analysis and machine learning methods. *Comb. Chem. High Throughput Screen.* **26**, 653–667 (2023). 35996248 10.2174/1386207325666220820163319

[59] Kim, S., Lin, C. W. & Tseng, G. C. MetaKTSP: a meta-analytic top scoring pair method for robust cross-study validation of omics prediction analysis. *Bioinformatics* **32**, 1966–1973 (2016). 27153719 10.1093/bioinformatics/btw115 PMC6280887

[60] Kaur, P., Schlatzer, D., Cooke, K. & Chance, M. R. Pairwise protein expression classifier for candidate biomarker discovery for early detection of human disease prognosis. *BMC Bioinform.* **13**, 191 (2012). 10.1186/1471-2105-13-191 PMC3468399 22870920

[61] Kernbach, J. M. & Staartjes, V. E. Foundations of machine learning-based clinical prediction modeling: Part II-Generalization and overfitting. *Acta Neurochir. Suppl.* **134**, 15–21 (2022). 34862523 10.1007/978-3-030-85292-4_3

[62] Clemente, F. M., Martins, F. M. L., Kalamaras, D., Wong, P. D. & Mendes, R. S. General network analysis of national soccer teams in FIFA World Cup 2014. *Int. J. Perform. Anal. Sport* **15**, 80–96 (2017).

[63] Wernicke, S. & Rasche, F. FANMOD: a tool for fast network motif detection. *Bioinformatics* **22**, 1152–1153 (2006). 16455747 10.1093/bioinformatics/btl038

[64] UniProt, C. UniProt: the universal protein knowledgebase in 2023. *Nucleic Acids Res.* **51**, D523–D531 (2023). 36408920 10.1093/nar/gkac1052 PMC9825514

[65] Giscard, P.-L., Richard, P. R. & Wilson, C. Evaluating balance on social networks from their simple cycles. *J. Complex Netw.* **5**, 750–775 (2017).

[66] Shannon, P. et al. Cytoscape: a software environment for integrated models of biomolecular interaction networks. *Genome Res.* **13**, 2498–2504 (2003). 14597658 10.1101/gr.1239303 PMC403769

[67] Holt, M. E. et al. My cancer genome: coevolution of precision oncology and a molecular oncology knowledgebase. *JCO Clin. Cancer Inf.* **5**, 995–1004 (2021). 10.1200/CCI.21.00084 PMC8807017 34554823

[68] Sheils, T. K. et al. TCRD and Pharos 2021: mining the human proteome for disease biology. *Nucleic Acids Res.* **49**, D1334–D1346 (2021). 33156327 10.1093/nar/gkaa993 PMC7778974

[69] Mendez, D. et al. ChEMBL: towards direct deposition of bioassay data. *Nucleic Acids Res.* **47**, D930–D940 (2019). 30398643 10.1093/nar/gky1075 PMC6323927

[70] Szalay-Beko, M. et al. ModuLand plug-in for Cytoscape: determination of hierarchical layers of overlapping network modules and community centrality. *Bioinformatics* **28**, 2202–2204 (2012). 22718784 10.1093/bioinformatics/bts352

[71] Lundberg, S. M. & Lee, S.-I. A unified approach to interpreting model predictions. In *31st Conference on Neural Information Processing Systems (NIPS 2017)* 4768–4777 (Curran Associates Inc., 2017).

[72] Alizadeh, E. A guide to Python environment, dependency and package management: Conda + Poetry. (2021).

[73] Gu, X. et al. Identification of dynamic network biomarker ITGB1 for erlotinib pre-resistance using single-cell differential covariance entropy. *Mol. Ther. Oncol.* **33**, 200993 (2025). 40546313 10.1016/j.omton.2025.200993 PMC12179664

[74] Matsuda, Y. et al. Combination of panobinostat with ponatinib synergistically overcomes imatinib-resistant CML cells. *Cancer Sci.* **107**, 1029–1038 (2016). 27166836 10.1111/cas.12965 PMC4946706

[75] Yang, G. et al. NDRG1 enhances the sensitivity to Cetuximab by promoting Stat1 ubiquitylation in colorectal cancer. *J. Adv. Res.* **72**, 555–569 (2025). 39128702 10.1016/j.jare.2024.07.035 PMC12147639

[76] Srivastava, R. M. et al. STAT1-induced HLA class I upregulation enhances immunogenicity and clinical response to anti-EGFR mAb cetuximab therapy in HNC patients. *Cancer Immunol. Res.* **3**, 936–945 (2015). 25972070 10.1158/2326-6066.CIR-15-0053 PMC4526378

[77] King, A. J. et al. Dabrafenib; preclinical characterization, increased efficacy when combined with trametinib, while BRAF/MEK tool combination reduced skin lesions. *PLoS ONE* **8**, e67583 (2013). 23844038 10.1371/journal.pone.0067583 PMC3701070

[78] Liu, L. et al. Sorafenib blocks the RAF/MEK/ERK pathway, inhibits tumor angiogenesis, and induces tumor cell apoptosis in hepatocellular carcinoma model PLC/PRF/5. *Cancer Res.* **66**, 11851–11858 (2006). 17178882 10.1158/0008-5472.CAN-06-1377

[79] Li, F. et al. Livin participates in resistance to trastuzumab therapy for breast cancer through ERK1/2 and AKT pathways and promotes EMT-like phenotype. *RSC Adv.* **8**, 28588–28601 (2018). 35542453 10.1039/c8ra05727c PMC9084334

[80] Pandya, K. et al. PKCalpha Attenuates Jagged-1-Mediated Notch Signaling in ErbB-2-Positive Breast Cancer to Reverse Trastuzumab Resistance. *Clin. Cancer Res.* **22**, 175–186 (2016). 26350262 10.1158/1078-0432.CCR-15-0179 PMC4703529

[81] Itah, Z. et al. HER2-driven breast cancer suppression by the JNK signaling pathway. *Proc. Natl. Acad. Sci. USA* **120**, e2218373120 (2023). 36656864 10.1073/pnas.2218373120 PMC9942916

[82] Xie, C. et al. PI3K/AKT/mTOR hypersignaling in autoimmune lymphoproliferative disease engendered by the epistatic interplay of Sle1b and FASlpr. *Int. Immunol.* **19**, 509–522 (2007). 17369192 10.1093/intimm/dxm017

[83] Kim, T. et al. Combinatorial CRISPR screen reveals FYN and KDM4 as targets for synergistic drug combination for treating triple negative breast cancer. *Elife* **13**, 10.7554/eLife.93921 (2025). 10.7554/eLife.93921 PMC12005726 40243589

[84] Stivala, S. et al. Targeting compensatory MEK/ERK activation increases JAK inhibitor efficacy in myeloproliferative neoplasms. *J. Clin. Investig.* **129**, 1596–1611 (2019). 30730307 10.1172/JCI98785 PMC6436863

