# Supplementary material for: MarkerPredict: predicting clinically relevant predictive biomarkers with machine learning
Source: NPJ Syst Biol Appl. 2025 Nov 21;11:132. doi: 10.1038/s41540-025-00603-0 (PMC12638940; doi:10.1038/s41540-025-00603-0)
Supplement: Supplementary file 1 — Supplementary Information [file 41540_2025_603_MOESM1_ESM.pdf]

Supplementary Material:

MarkerPredict: ***predicting clinically relevant predictive biomarkers with machine learning***

Daniel V. Veres[1,2], Peter Csermely[1], Klára Schulc[1,3]

[1] Semmelweis University, Department of Molecular Biology, Budapest 1085, Hungary

[2] Turbine Ltd., Budapest, Hungary

[3] Division of Oncology, Department of Internal Medicine and Oncology, Semmelweis University, Budapest, Hungary

Contents

Supplementary Material: 1

Supplementary Text S1 | Hyperparameters used for the machine learning models.................. 5

Supplementary Text S2 | The MarkerPredict Python-package on GitHub 7

Supplementary Figure S1 | The ratio of different motif types in the networks 8

Supplementary Figure S2 | Correlation between different IDP databases 9

a) DisProt disorder % - AlphaFold disorder % 9

b) DisProt disorder % - IUPred long disorder %.. 10

c) DisProt disorder % - IUPred short disorder % 11

d) AlphaFold disorder % - IUPred long disorder % 12

e) AlphaFold disorder % - IUPred short disorder % 13

Supplementary Figure S3 | Results of 70:30 train:test split with CIViCmine-defined and random negative training sets 14

a) ROC curve of CIViCmine-defined negative control set 14

b) ROC curve of random negative control set 15

Supplementary Figure S4 | Accuracy values of the models trained on different input parameters 16

Supplementary Figure S5 | Metrics after cross-training of the models 17

a) Cross-training with both biological and topological data (all databases) 18

b) Cross-training with both biological and topological data (DisProt) 19

c) Cross-training with both biological and topological data (AlphaFold) 20

d) Cross-training with both biological and topological data (IUPred) 21

e) Cross-training with only topological data (all databases) 22

f) Cross-training with only topological data (DisProt) 23

g) Cross-training with only topological data (AlphaFold) 24

h) Cross-training with only topological data (IUPred) 25

Supplementary Figure S6 | The results of SHAP analysis 26

a) all databases – all networks - XGBoost 27

b) all databases – all networks - Random Forest 28

c) DisProt – all networks – XGBoost 29

d) DisProt – all networks - Random Forest 30

e) AlphaFold – all networks – XGBoost 31

f) AlphaFold – all networks - Random Forest 32

g) IUPred – all networks – XGBoost 33

h) IUPred – all networks - Random Forest 34

Supplementary Figure S7 | A known biomarker also predicted by the MarkerPredict framework: Notch1 as a potential predictive biomarker for HDAC-inhibitors 35

Supplementary Figure S8 | A new biomarker predicted by the MarkerPredict framework: β-catenin as a potential predictive biomarker for ponatinib 37

Supplementary Figure S9 | A known biomarker also predicted by the MarkerPredict framework: CREB1 as a potential predictive biomarker for HER2-inhibitors... 39

Supplementary Figure S10 | A new biomarker predicted by the MarkerPredict framework: Integrin β1 as a potential predictive biomarker for c-Met inhibitor cabozantinib 41

Supplementary Table S1 | Topological parameters of the three signalling networks used in the study 44

Supplementary Table S2 | The input dataset of the machine learning model *(available on GitHub)* 45

Supplementary Table S3 | The input parameters of the machine learning model 45

Supplementary Table S4 | Performance of the machine learning models with different validation methods 48

Supplementary Table S5 | The results of the final prediction *(available on GitHub)* 52

Supplementary References 53

Supplementary Text S1 | Hyperparameters used for the machine learning models

The acquired hyperparameters of competitive random halving *via* the scikit-learn package[1].

```
xgb = xgb_package.XGBClassifier(n_estimators=1000,
                                random_state=42,
                                subsample= 0.75,
                                scale_pos_weight= 0.25,
                                reg_lambda= 0,
                                reg_alpha= 0.5,
                                min_child_weight= 0.5,
                                max_leaves= 11,
                                max_depth= 7,
                                max_delta_step= 1,
                                max_bin= 32,
                                learning_rate= 1,
                                importance_type= 'cover',
                                grow_policy= 'lossguide',
                                gamma= 0.5,
                                criterion= 'log_loss',
                                booster= 'gbtree')
rfc = RandomForestClassifier(n_estimators=1000,
                             random_state= 42,
                             warm_start= False,
                             oob_score= False,
                             n_jobs= 31,
                             min_weight_fraction_leaf= 0.1,
                             min_samples_split= 4,
                             min_samples_leaf= 5,
                             max_samples= 71,
                             max_leaf_nodes= 29,
                             max_features= 'sqrt',
                             max_depth= 3,
                             criterion= 'log_loss',
                             class_weight= 'balanced',
                             ccp_alpha= 0)
```

Supplementary Text S2 | The MarkerPredict Python-package on GitHub

To share the code created in our project, we established a Python package on GitHub. The separate validating and predicting processes can be found in separate Jupyter Notebooks. The dependency list is created and can be implemented with the *poetry* package. Necessary datasets are also uploaded in the form of CSV files.

The link to our repository:

https://github.com/klari98/MarkerPredict

Supplementary Figure S1 | The ratio of different motif types in the networks

he number of cycles and unbalanced triangles in triangles with and without DisProt IDP-target pairs in the Human Cancer Signaling Network (CSN), SIGNOR and ReactomeFI (with logarithmic scale). The significance levels of the performed chi-square tests are highlighted on the figures.

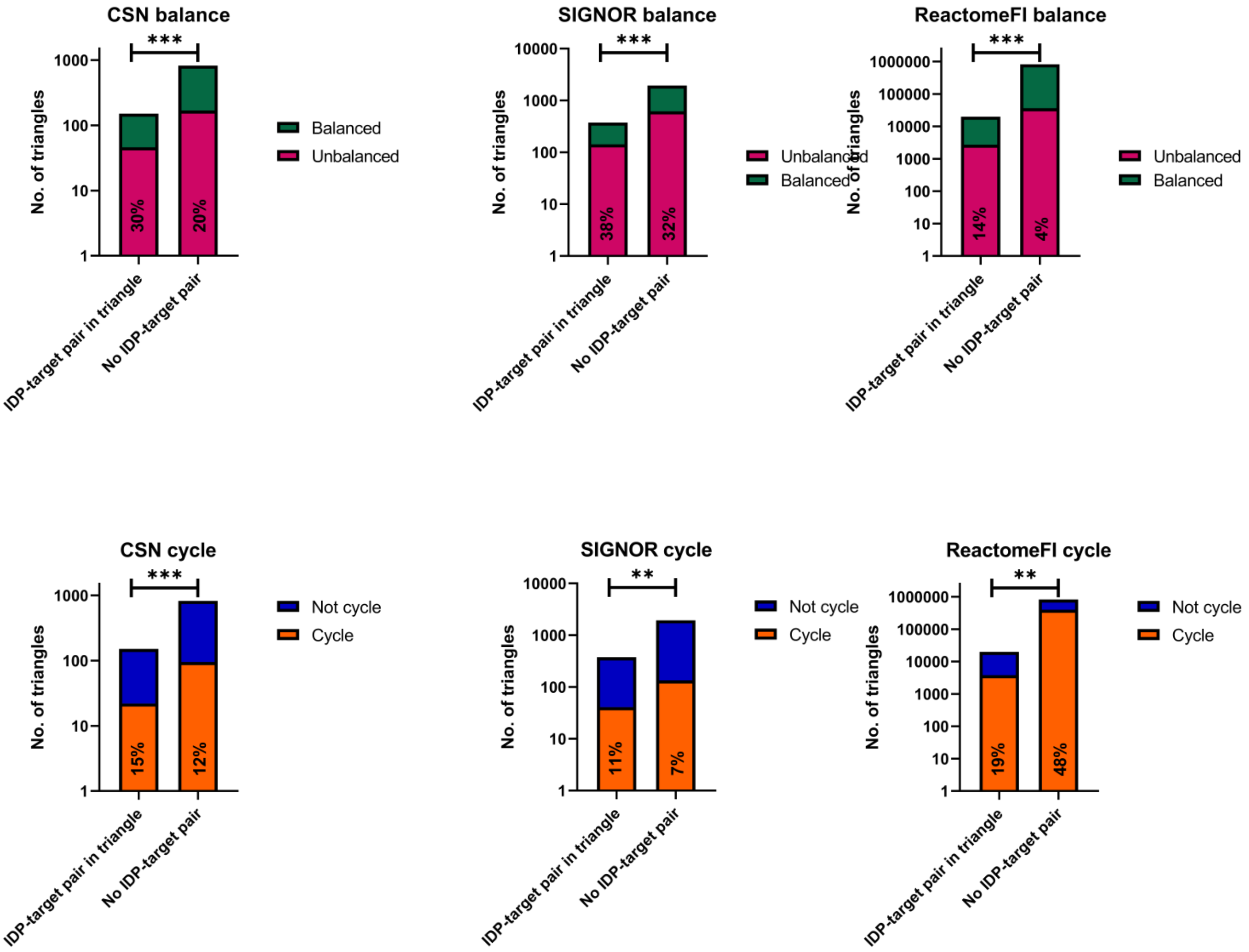

Supplementary Figure S2 | Correlation between different IDP databases

a) DisProt disorder % - AlphaFold disorder %

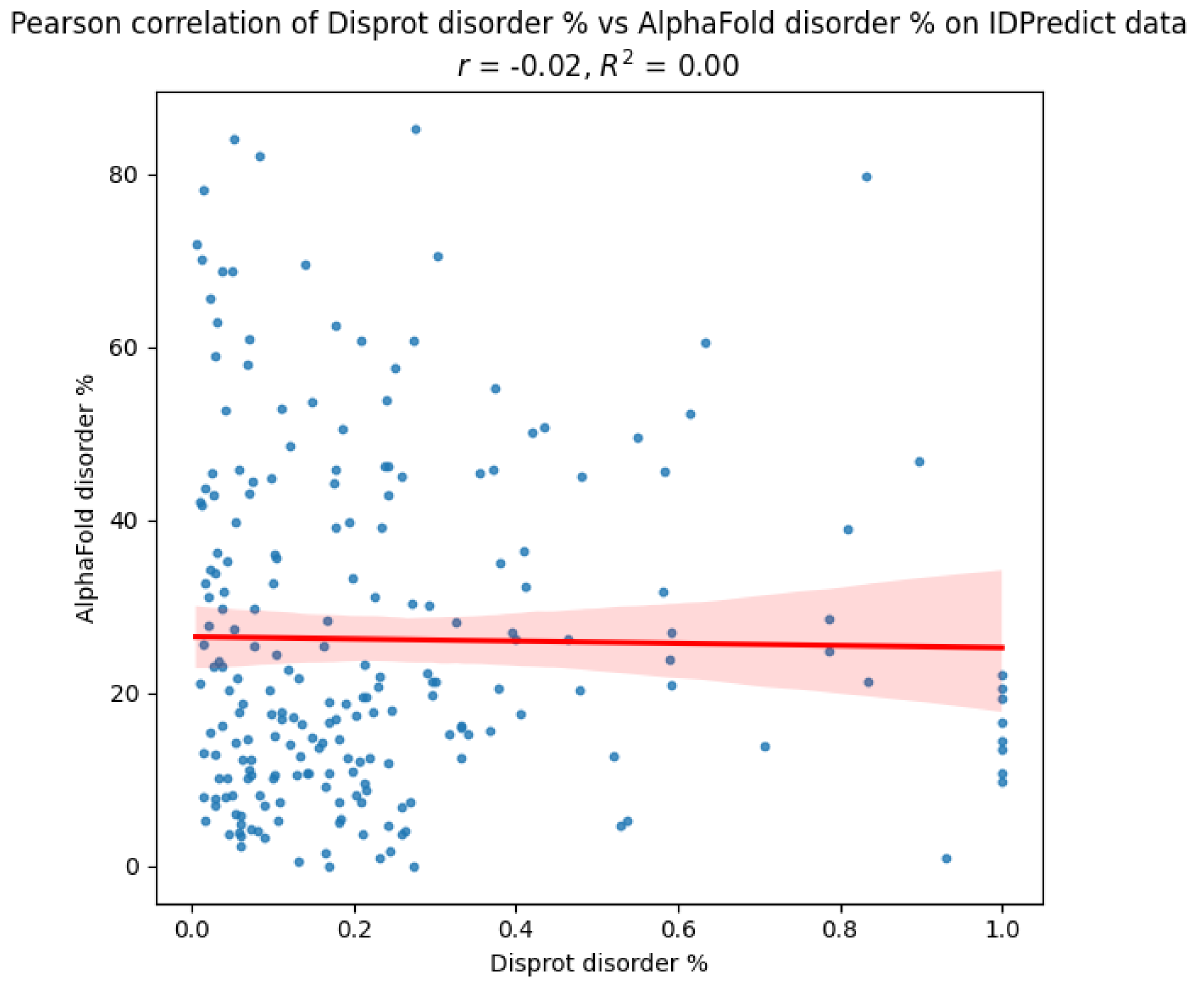

b) DisProt disorder % - IUPred long disorder %

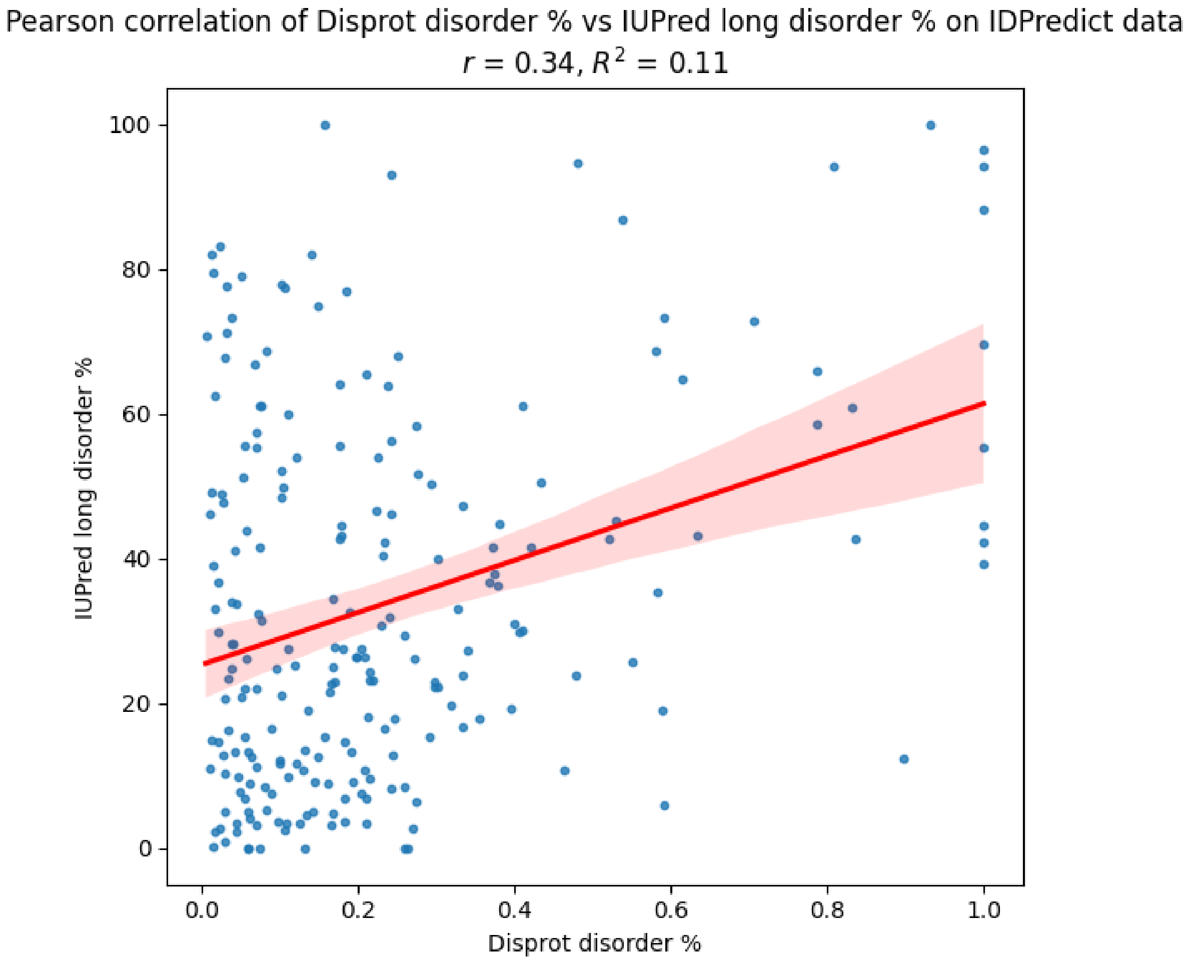

c) DisProt disorder % - IUPred short disorder %

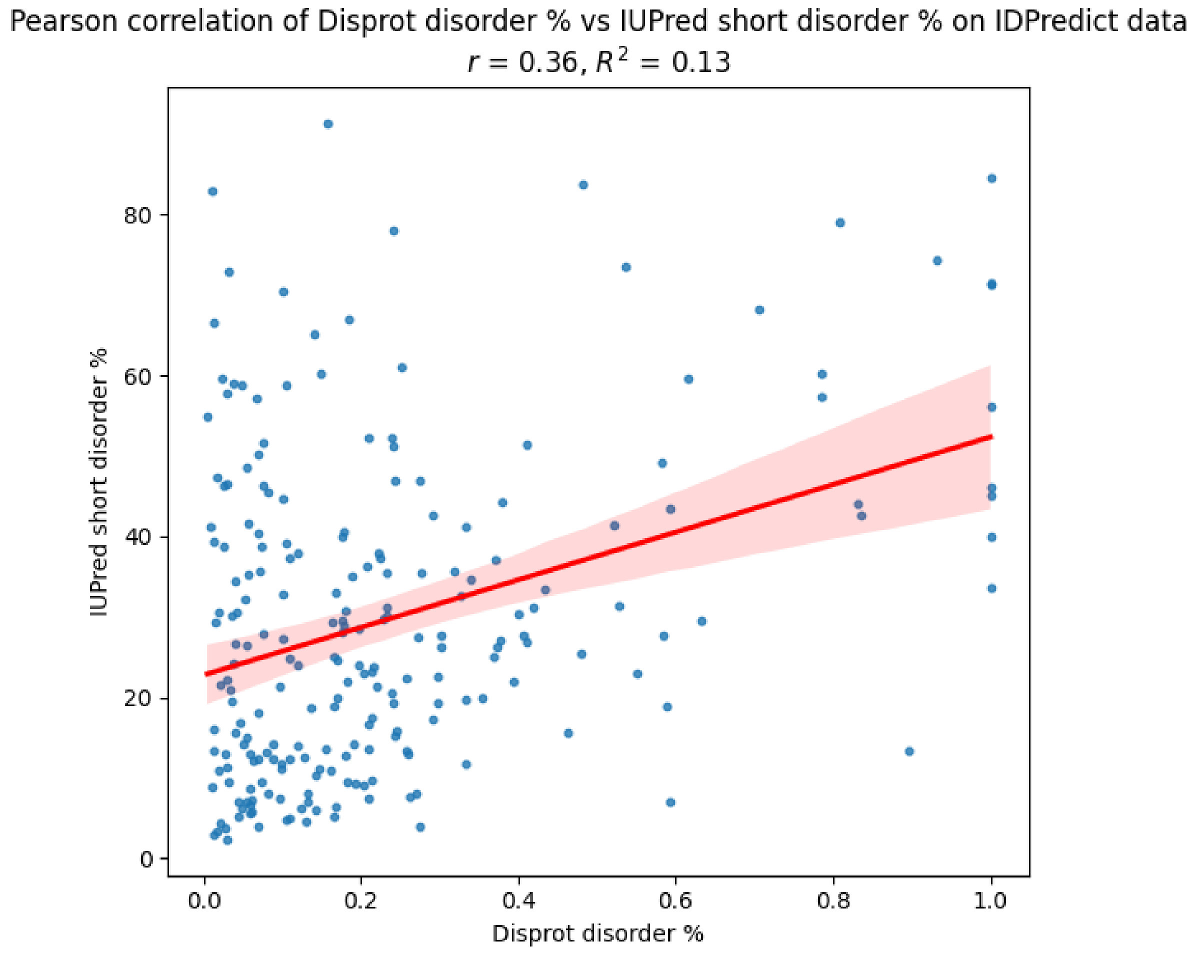

d) AlphaFold disorder % - IUPred long disorder %

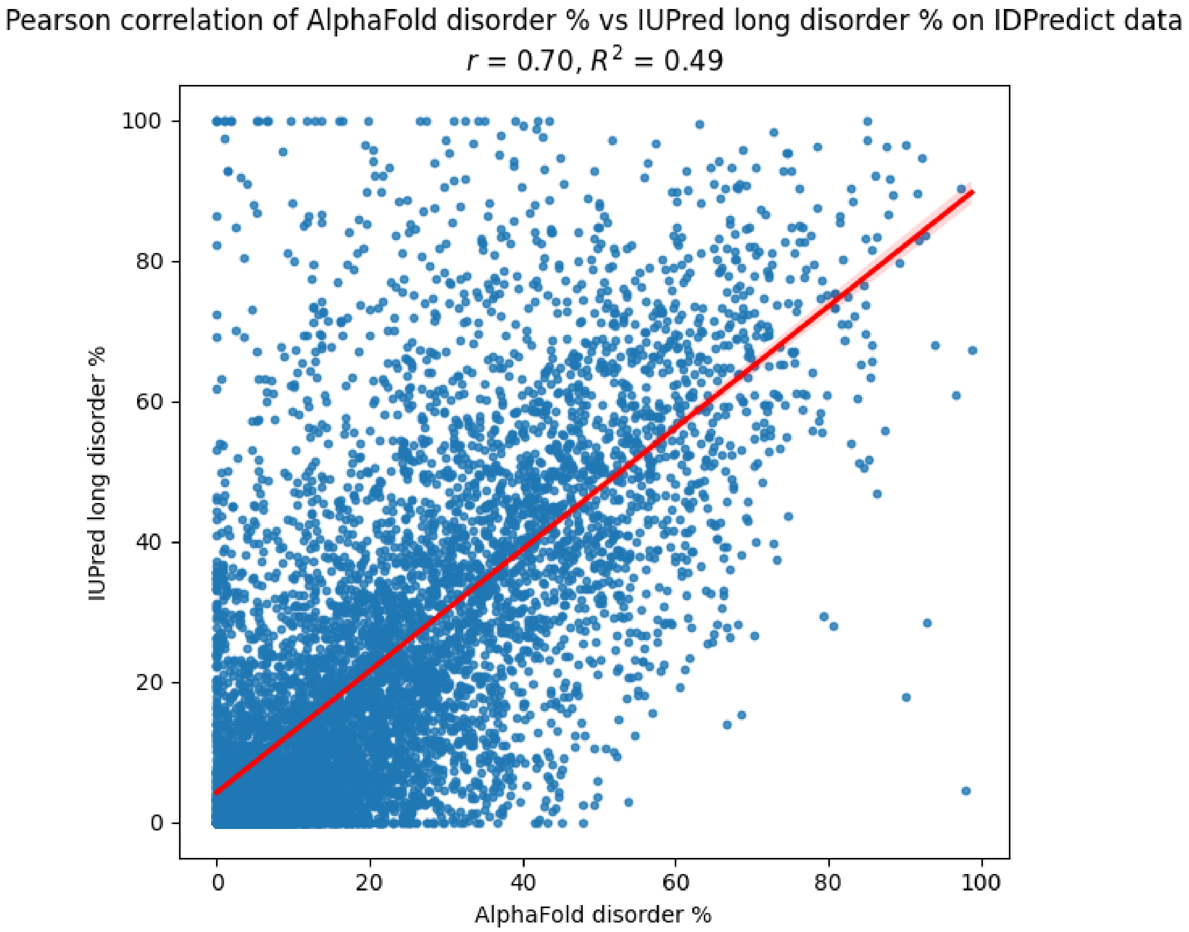

e) AlphaFold disorder % - IUPred short disorder %

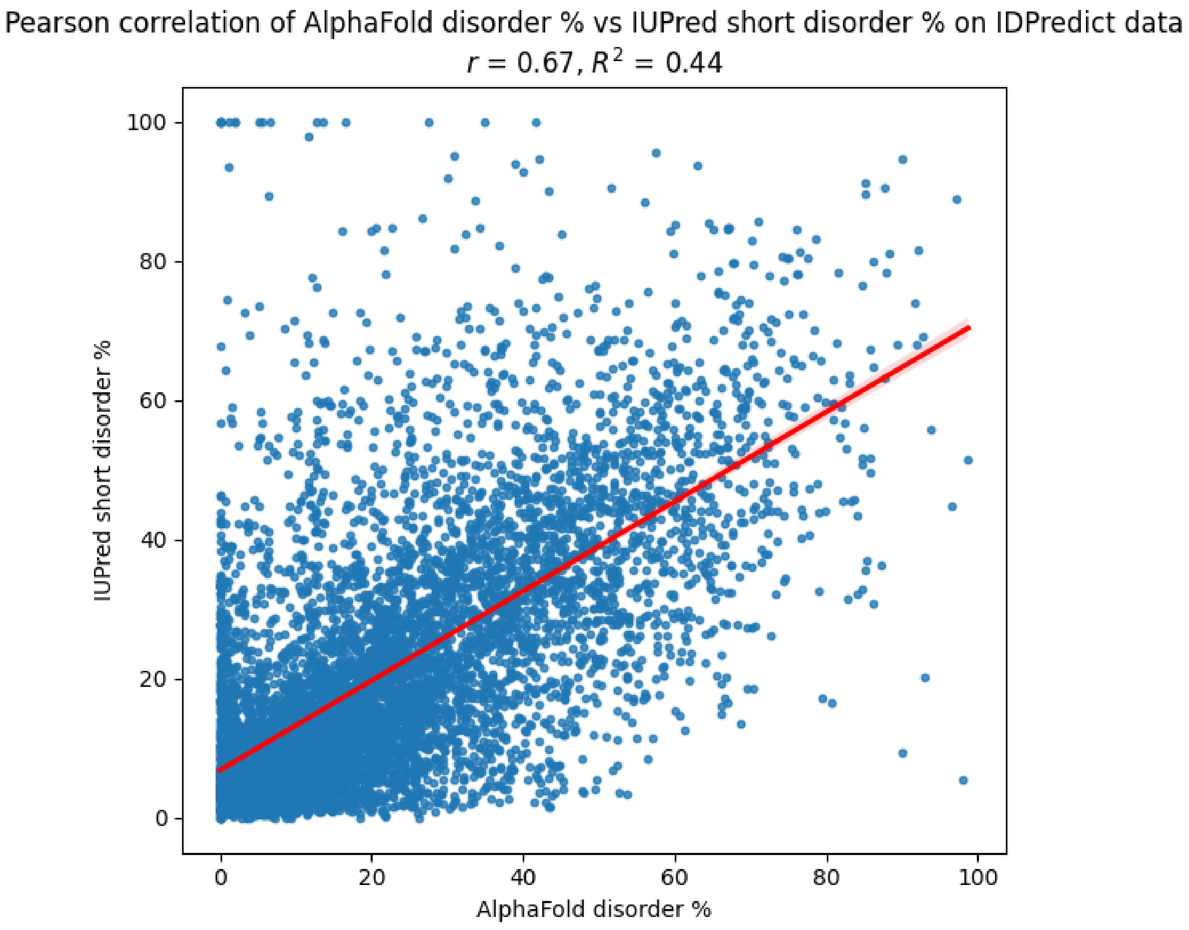

Supplementary Figure S3 | Results of 70:30 train:test split with CIViCmine-defined and random negative training sets

a) ROC curve of CIViCmine-defined negative control set

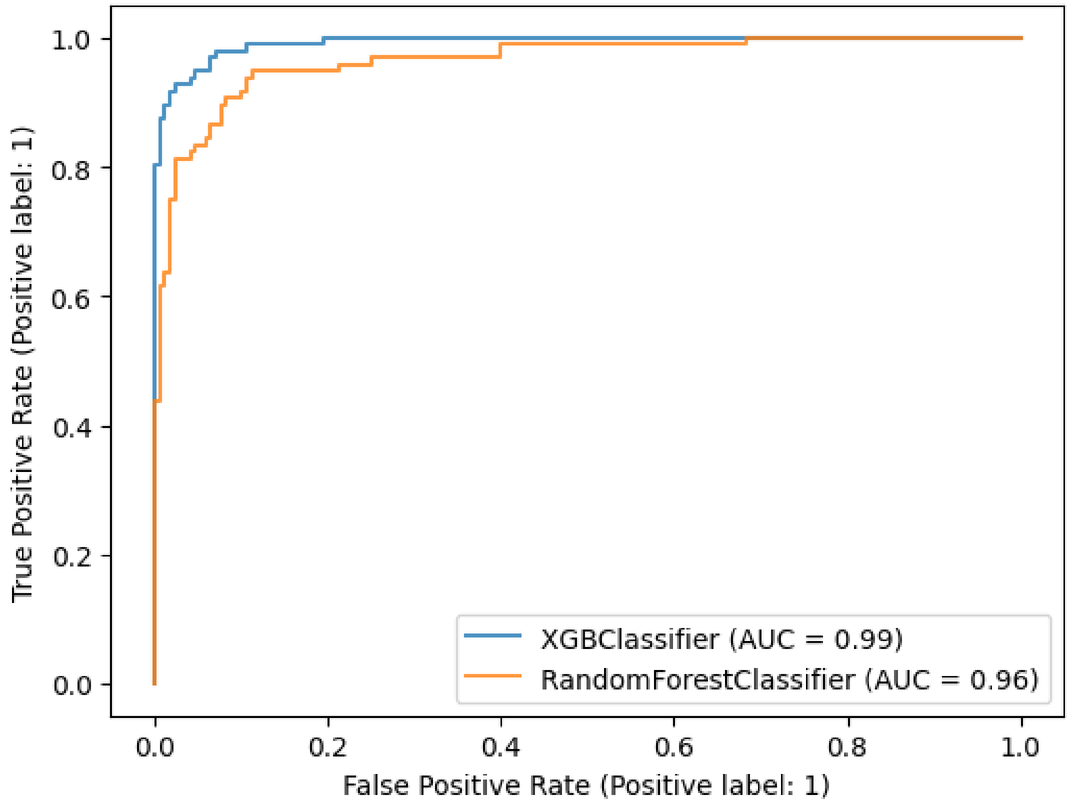

b) ROC curve of random negative control set

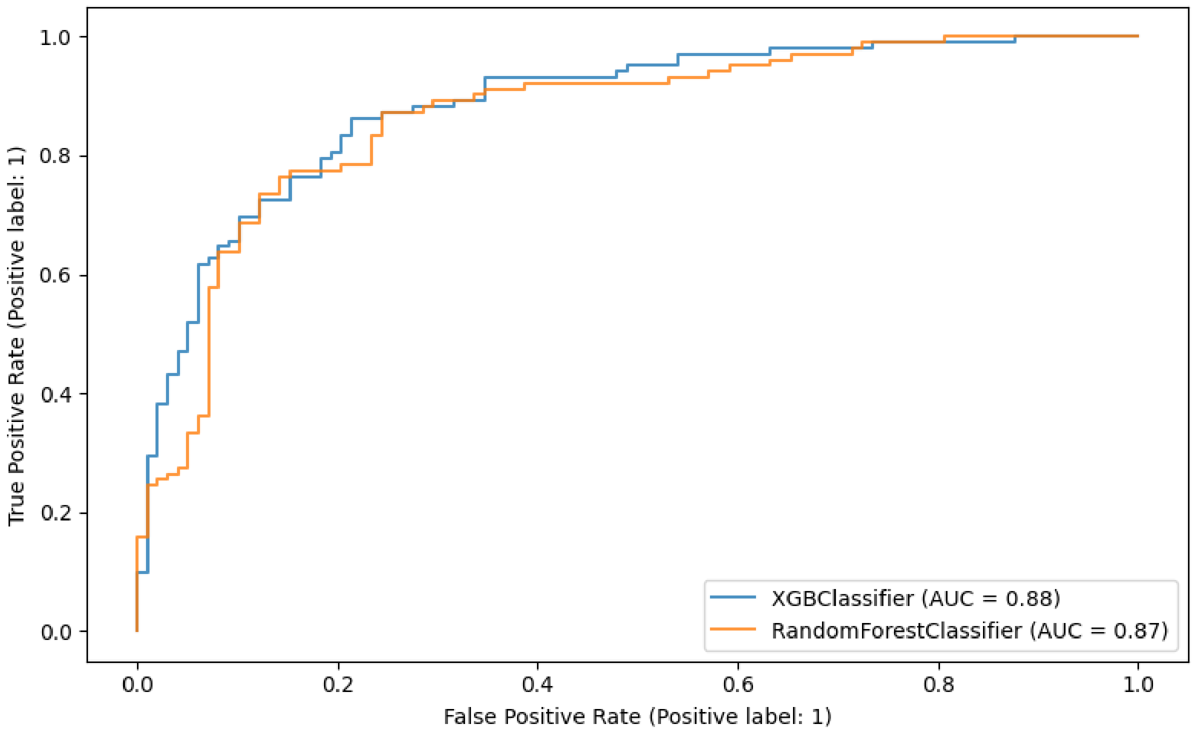

Supplementary Figure S4 | Accuracy values of the models trained on different input parameters

We trained the machine learning models on different group of input parameters. The models reached good metrics on both biological and topological parameters.

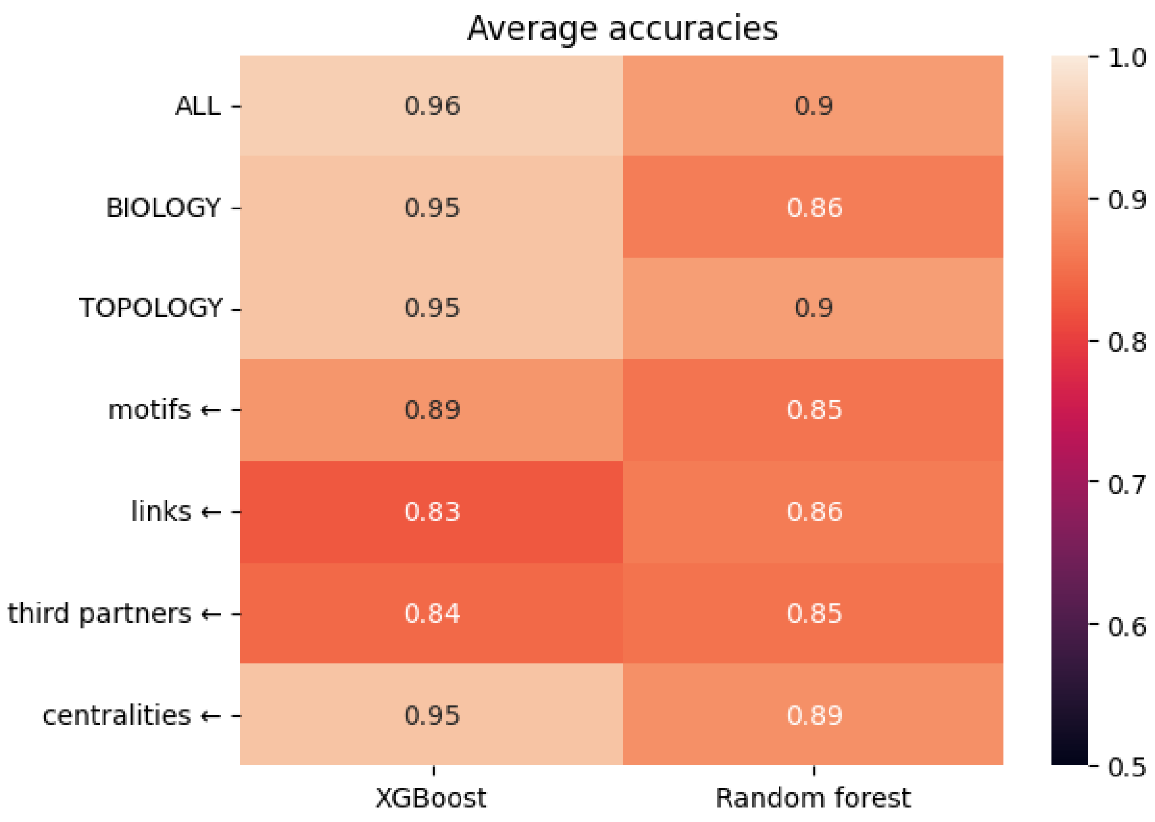

Supplementary Figure S5 | Metrics after cross-training of the models

We cross-trained among networks, i.e., we trained on the data of one network and tested on the other.

a) Cross-training with both biological and topological data (all databases)

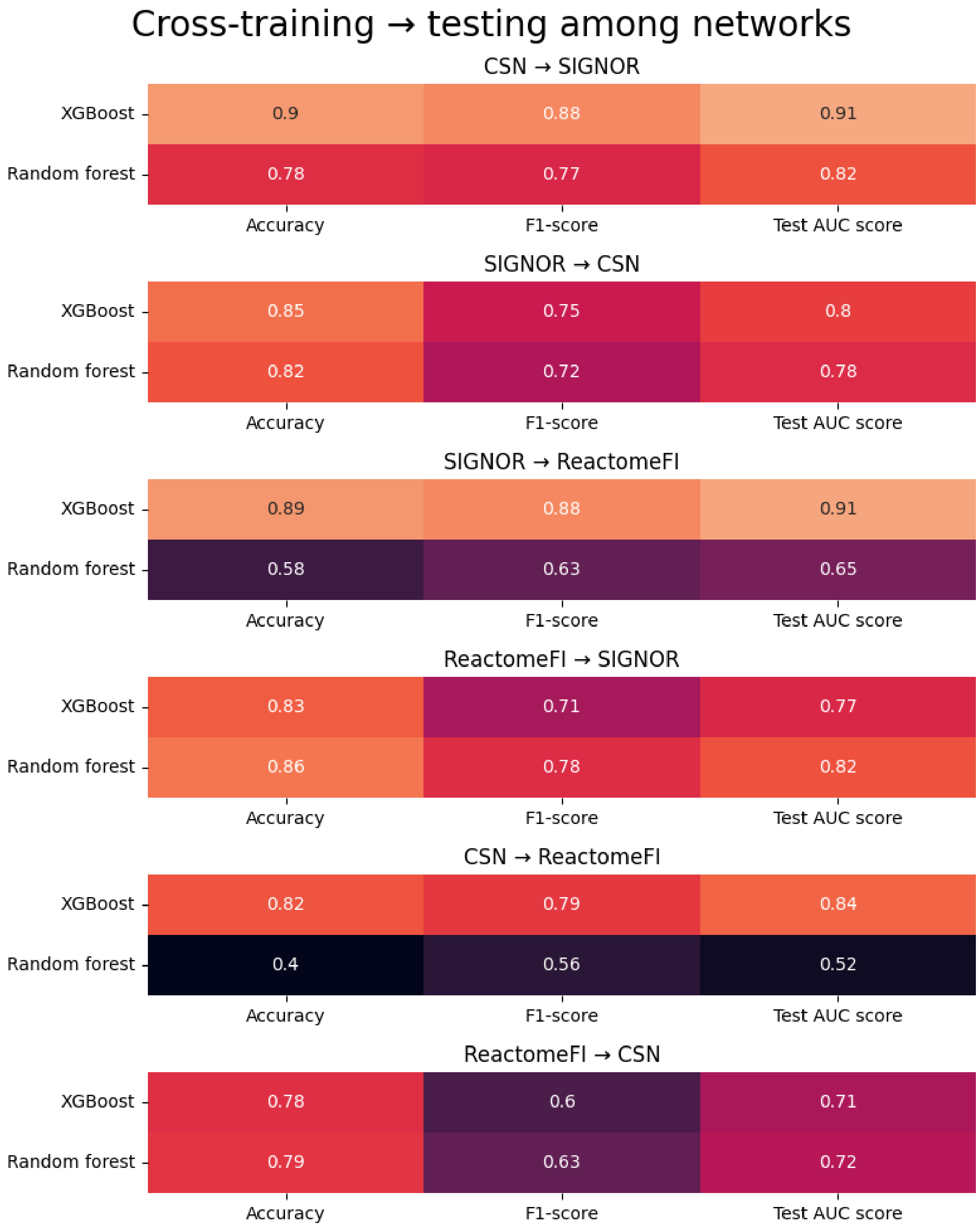

b) Cross-training with both biological and topological data (DisProt)

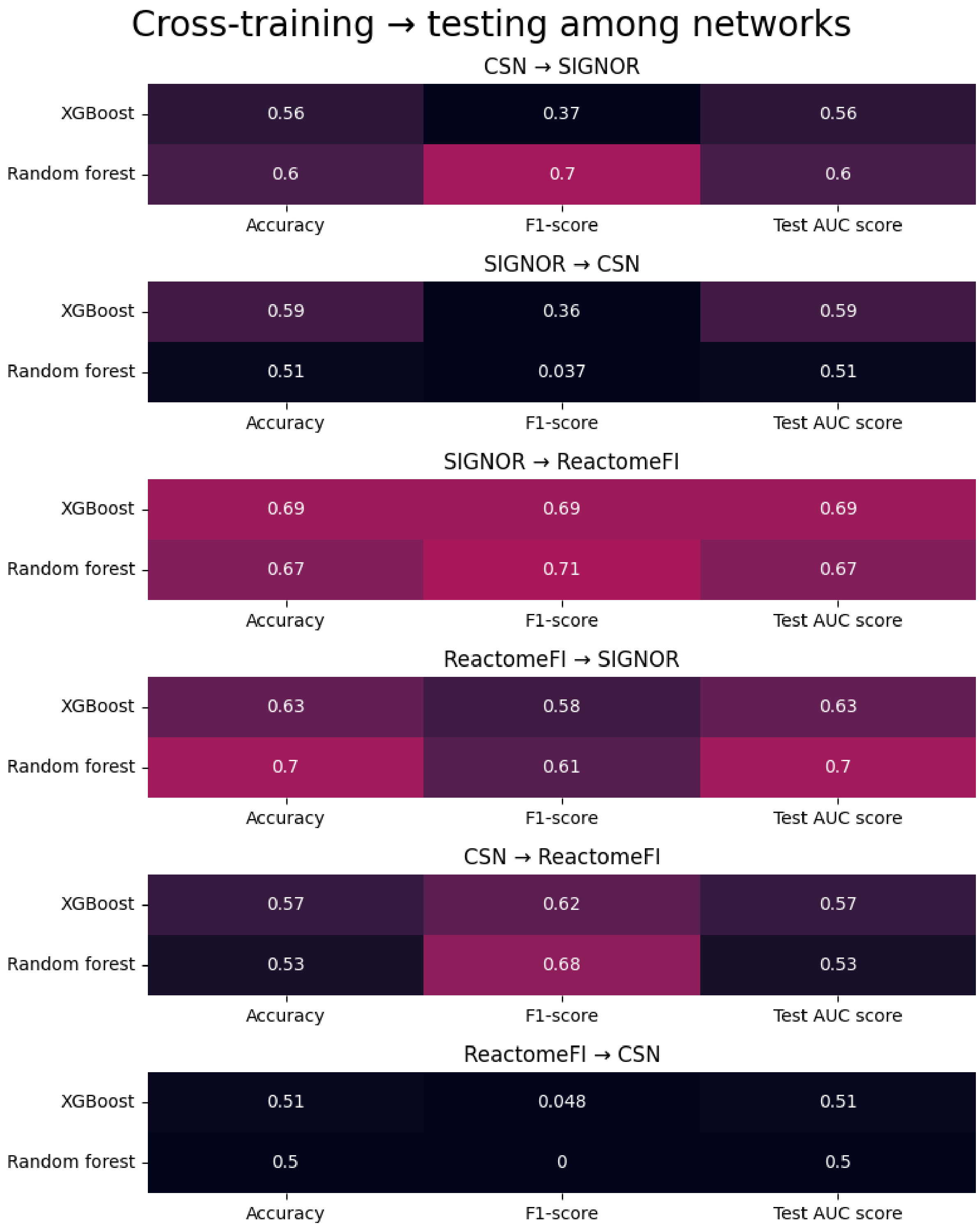

c) Cross-training with both biological and topological data (AlphaFold)

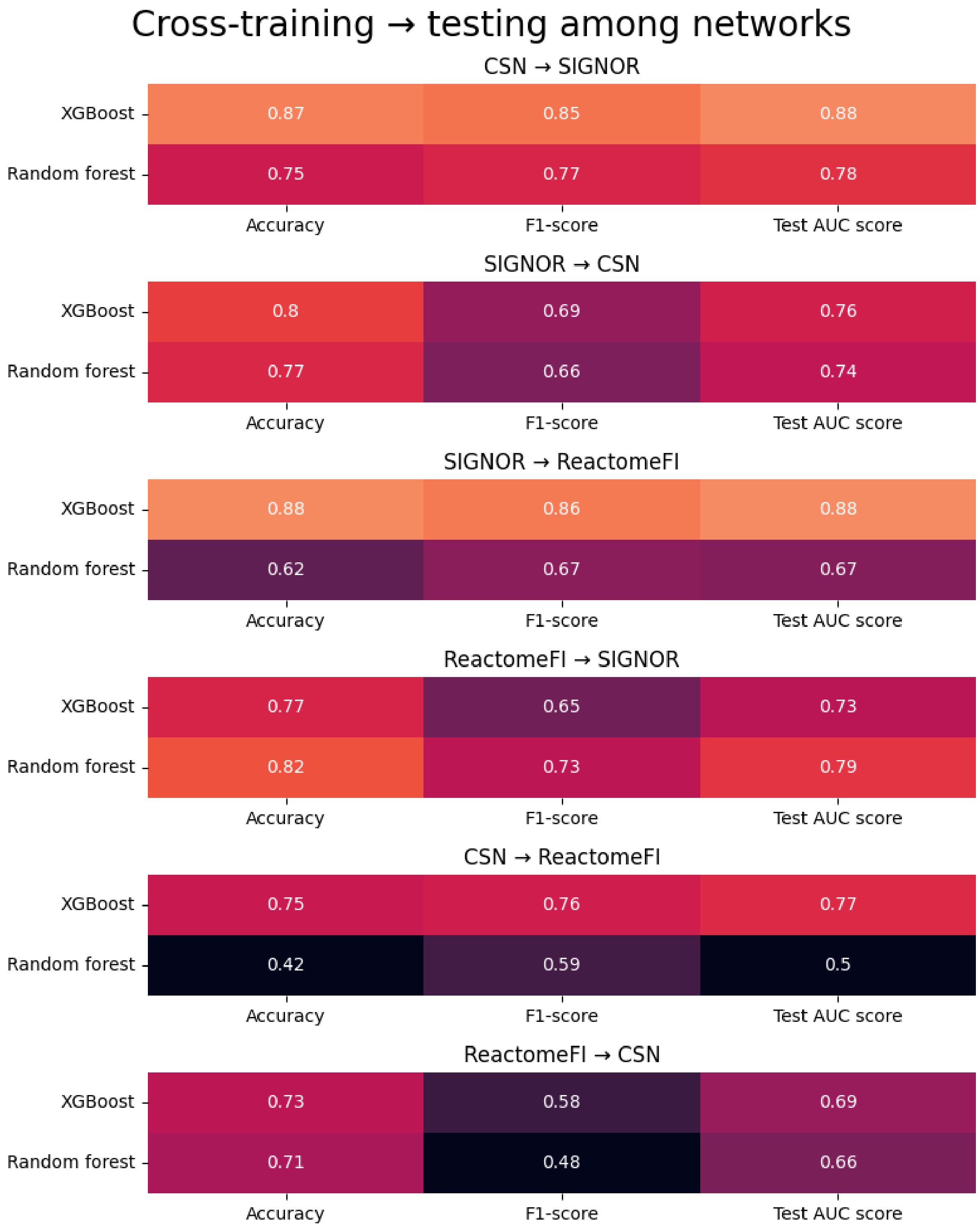

d) Cross-training with both biological and topological data (IUPred)

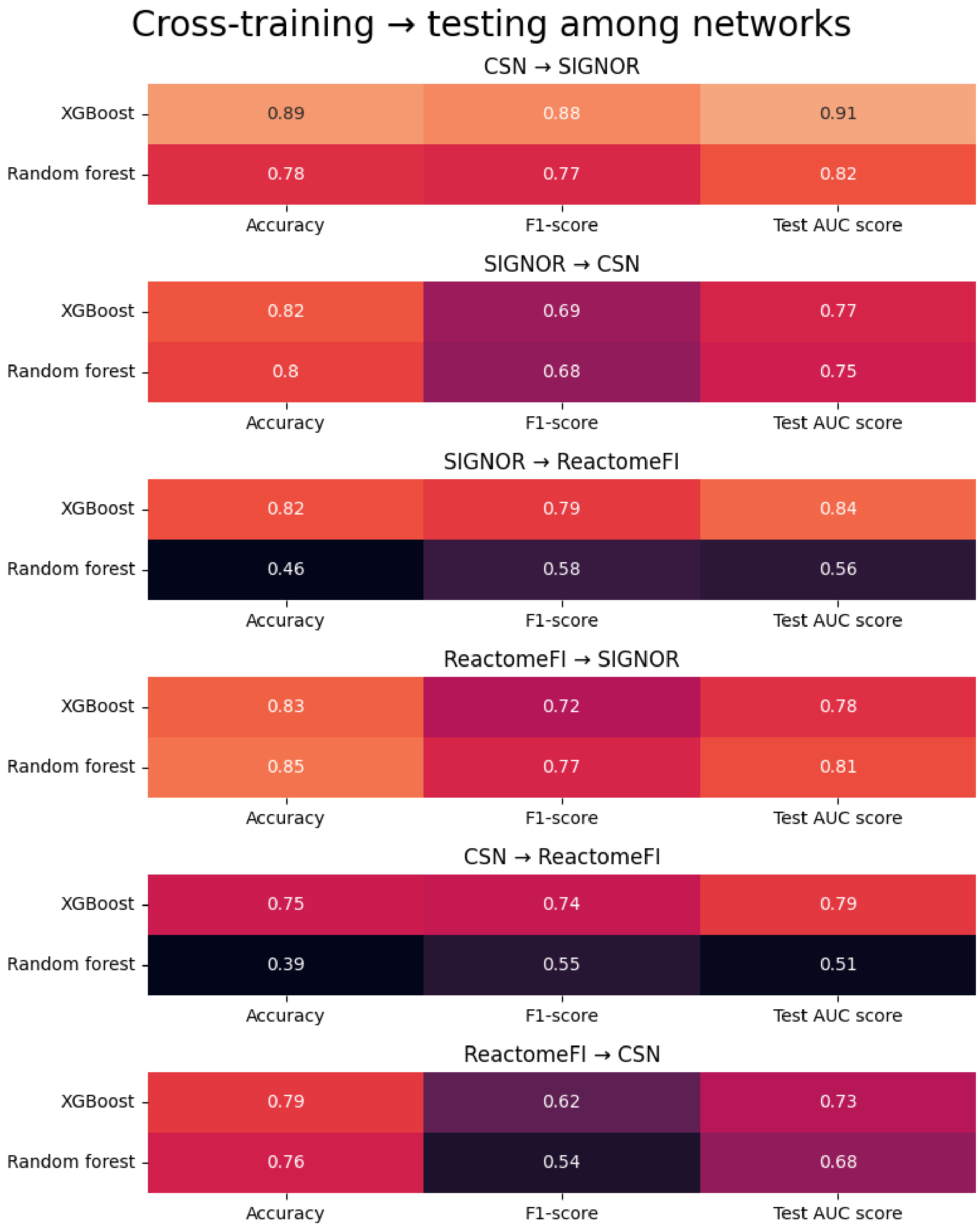

e) Cross-training with only topological data (all databases)

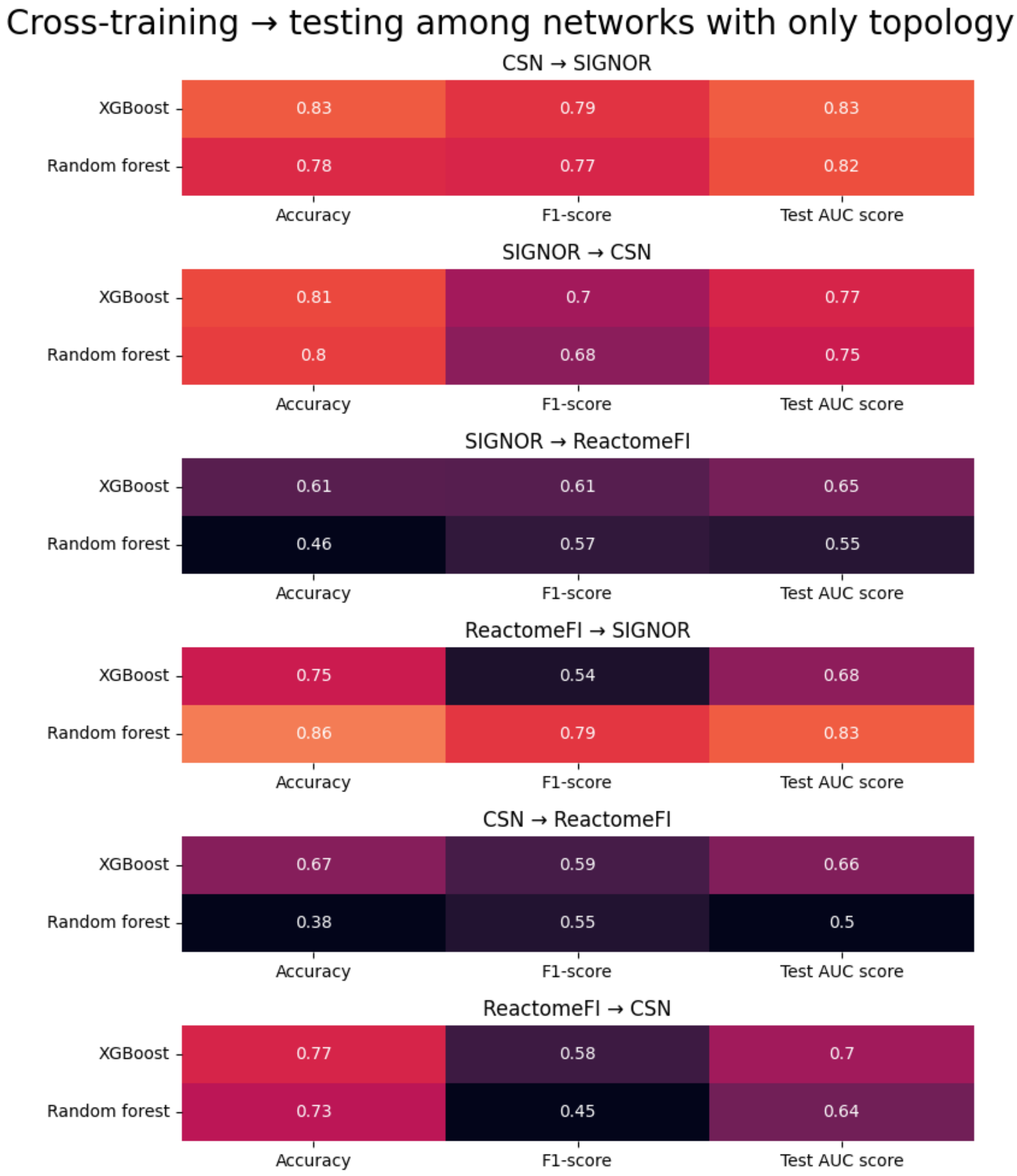

f) Cross-training with only topological data (DisProt)

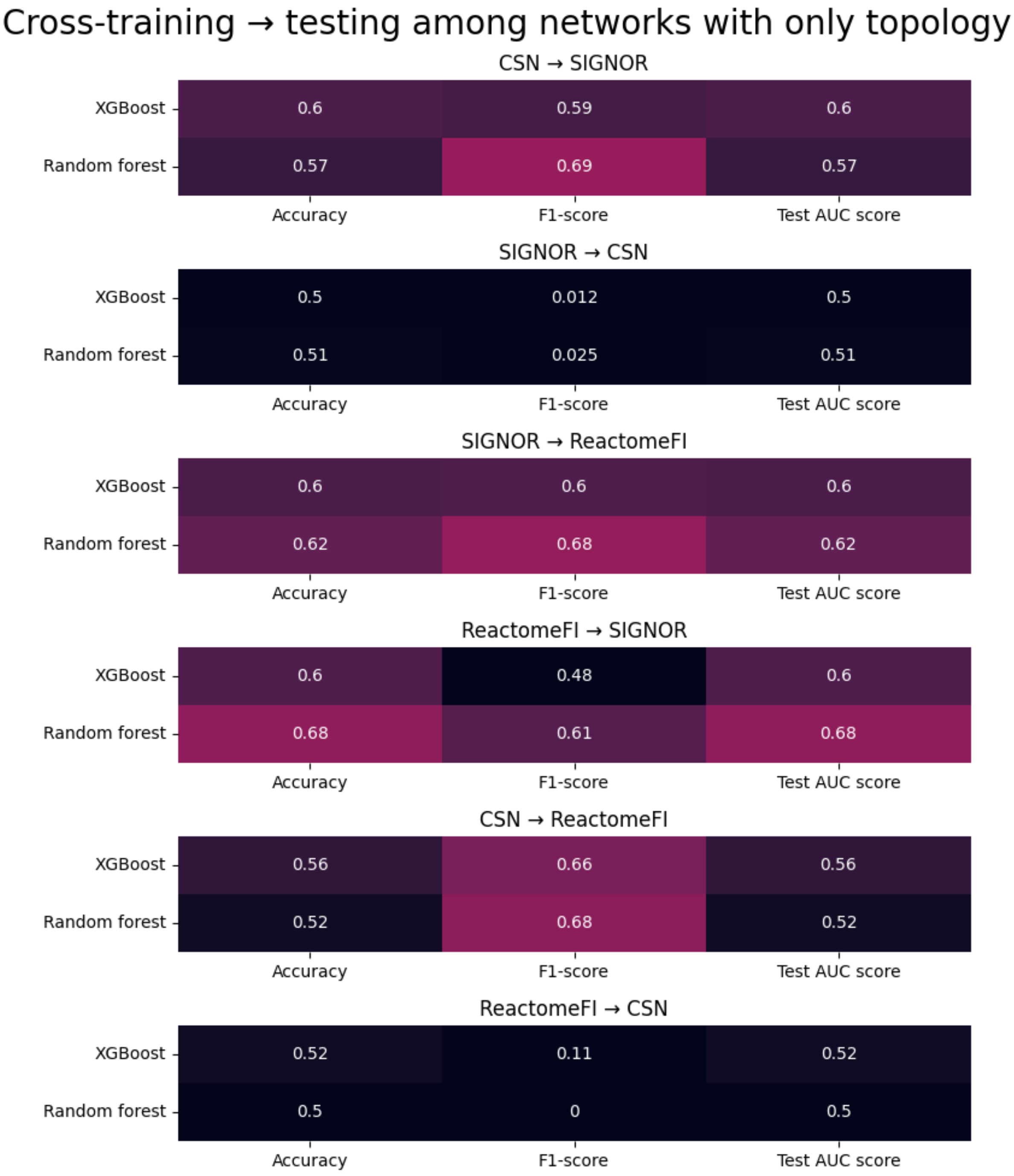

g) Cross-training with only topological data (AlphaFold)

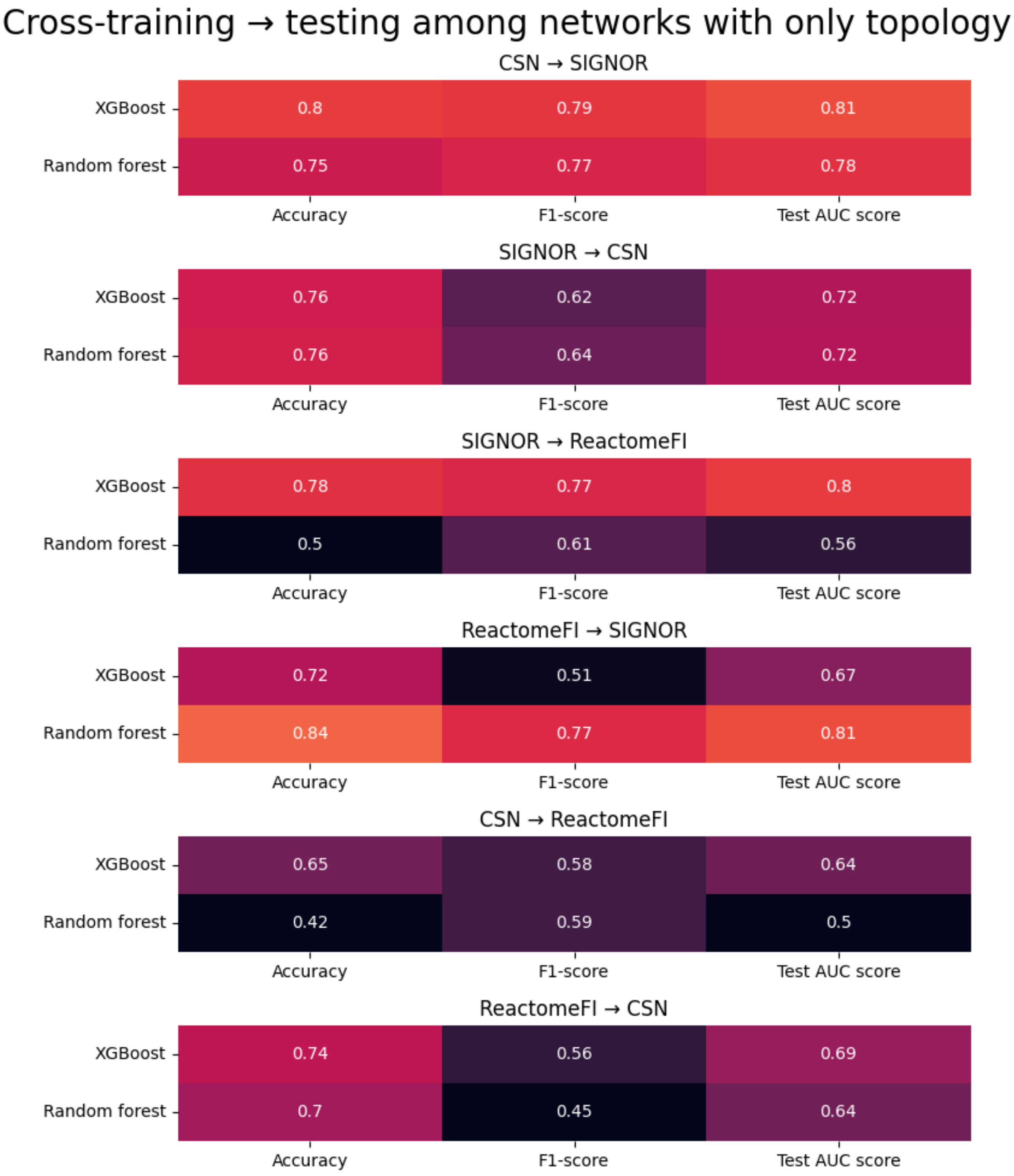

h) Cross-training with only topological data (IUPred)

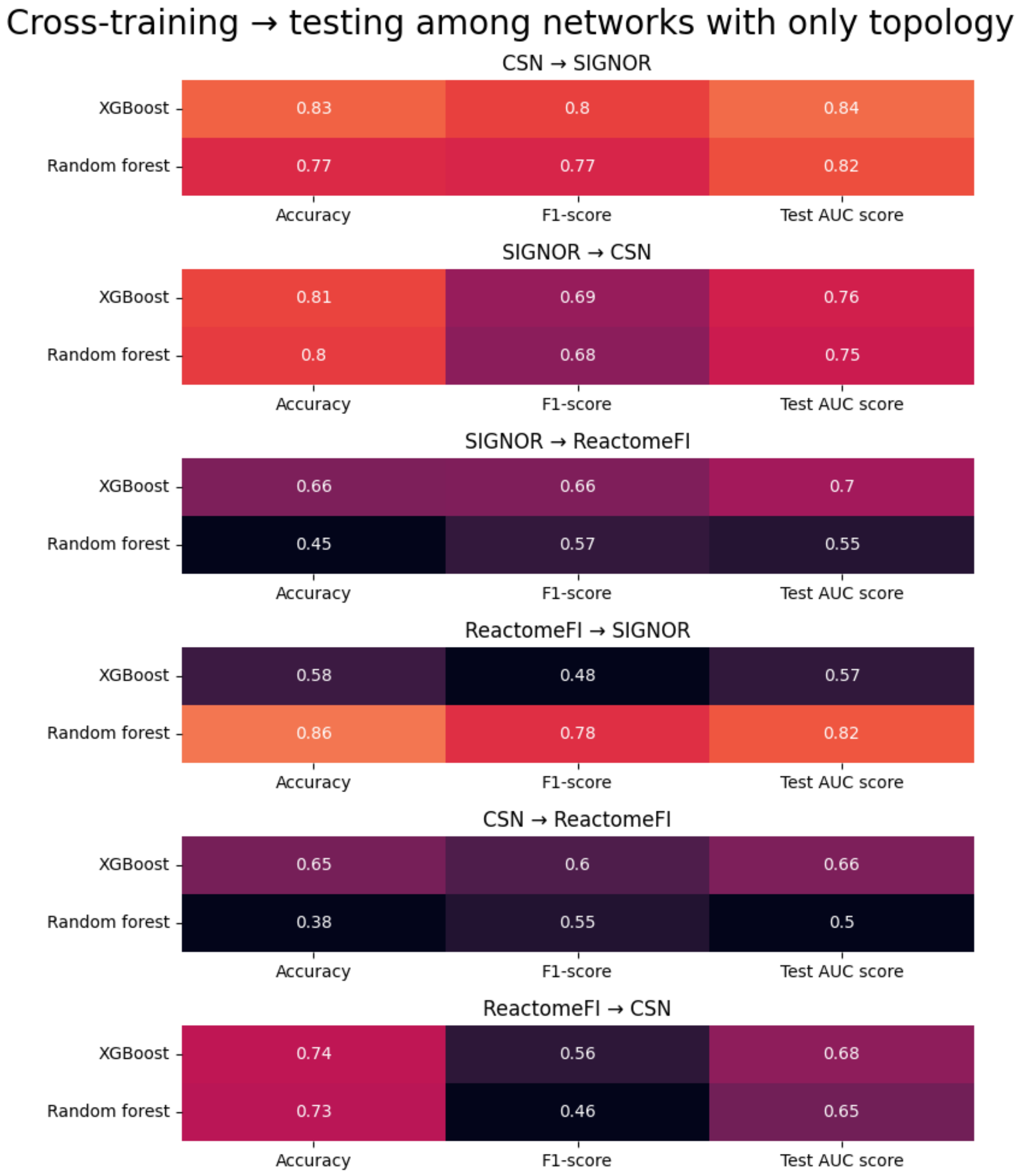

Supplementary Figure S6 | The results of SHAP analysis

We made a SHAP analysis[2] of the classification based on the data of different IDP databases, such as DisProt, AlphaFold and IUPred. The most important parameters had a large overlap. Disorder content was an important parameter for all the databases.

a) all databases – all networks - XGBoost

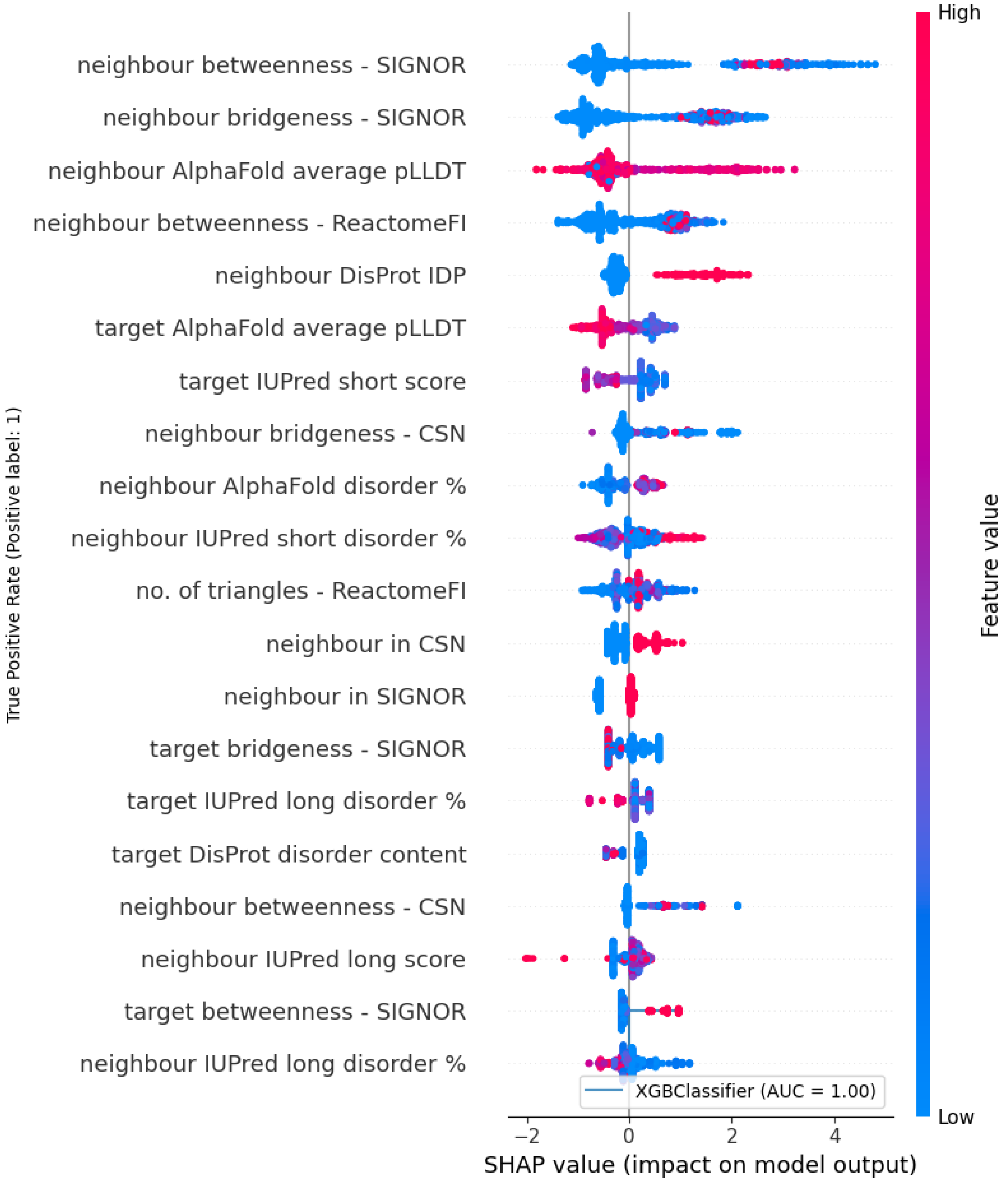

b) all databases – all networks - Random Forest

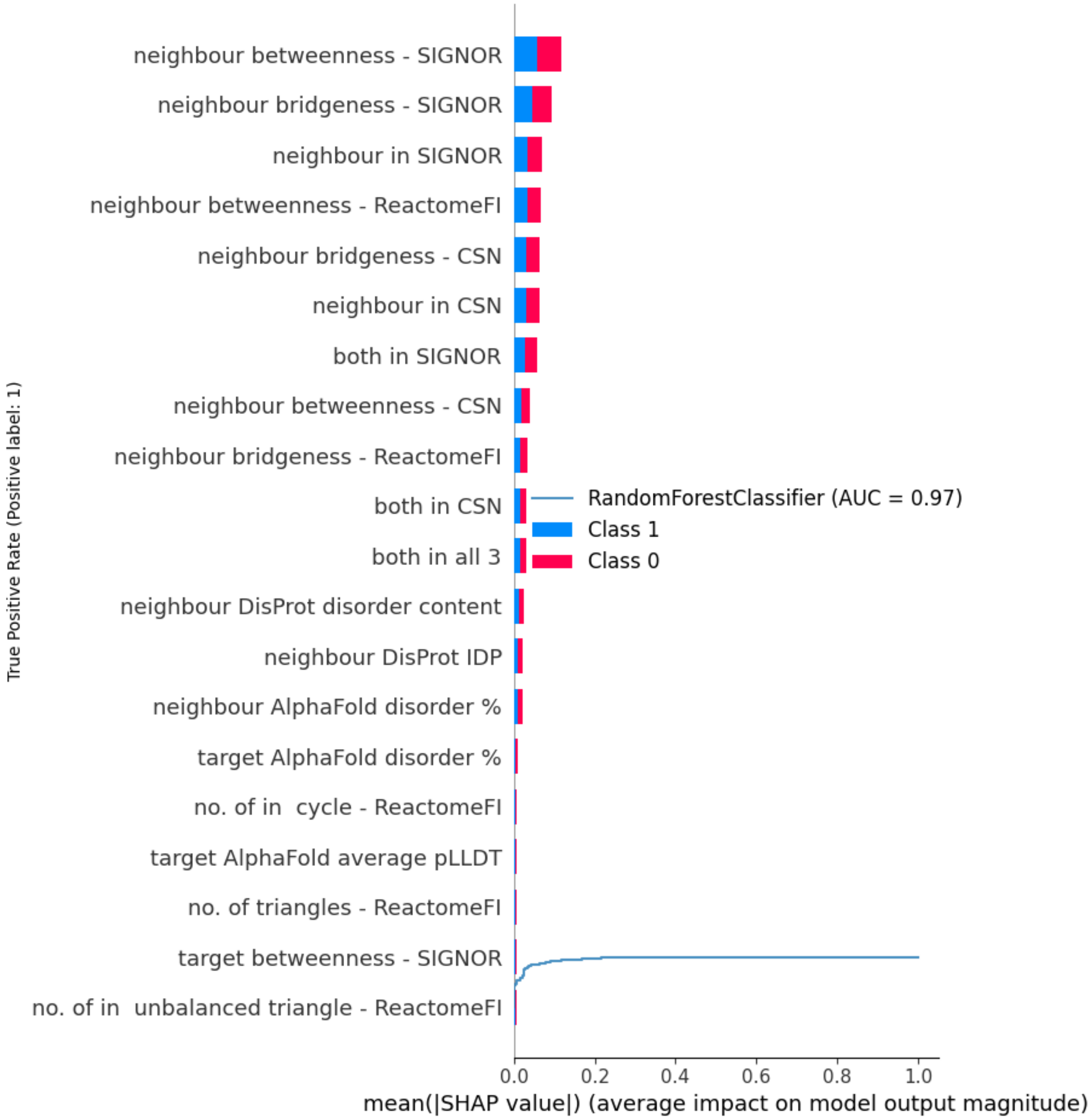

c) DisProt – all networks – XGBoost

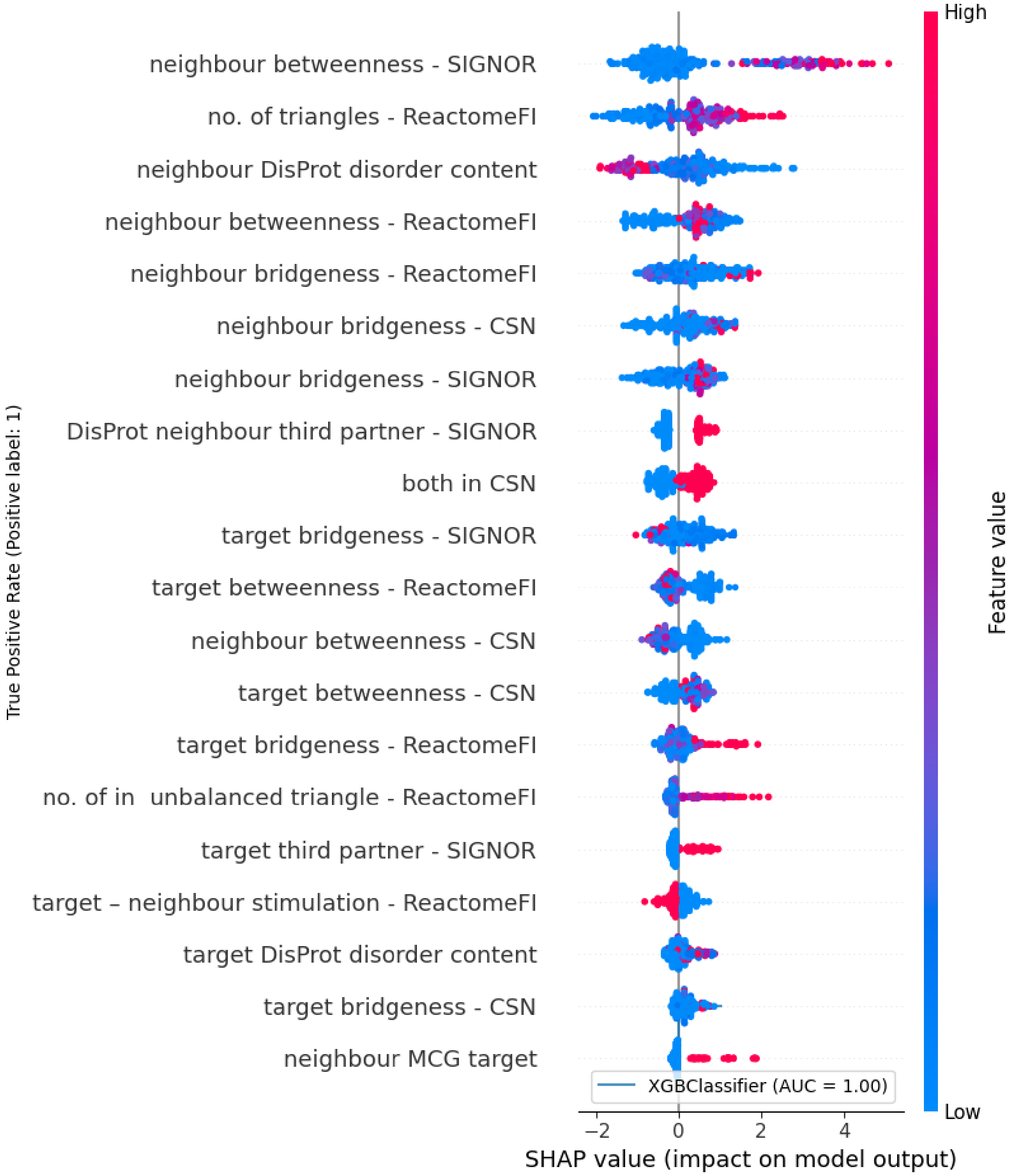

d) DisProt – all networks - Random Forest

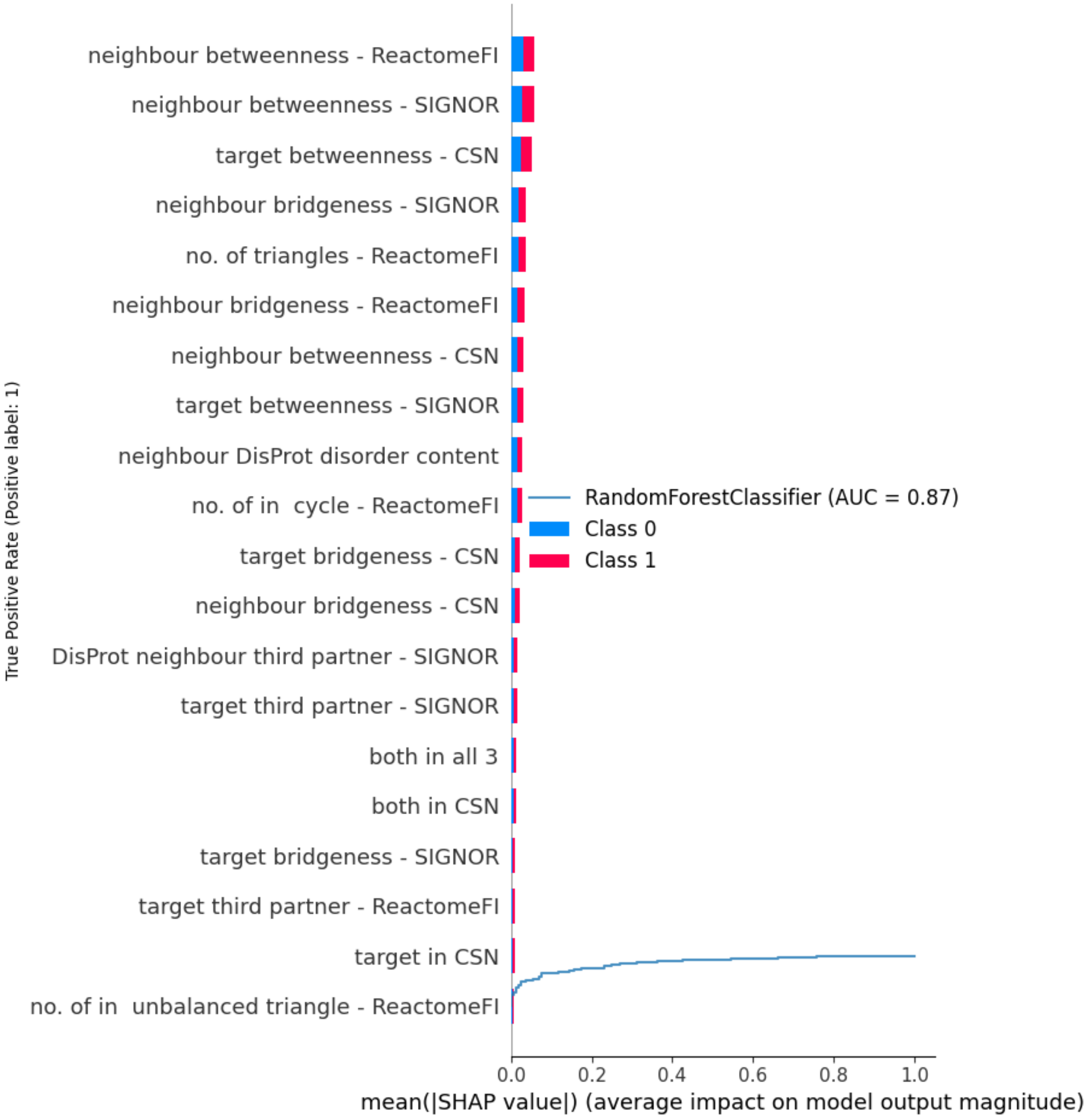

e) AlphaFold – all networks – XGBoost

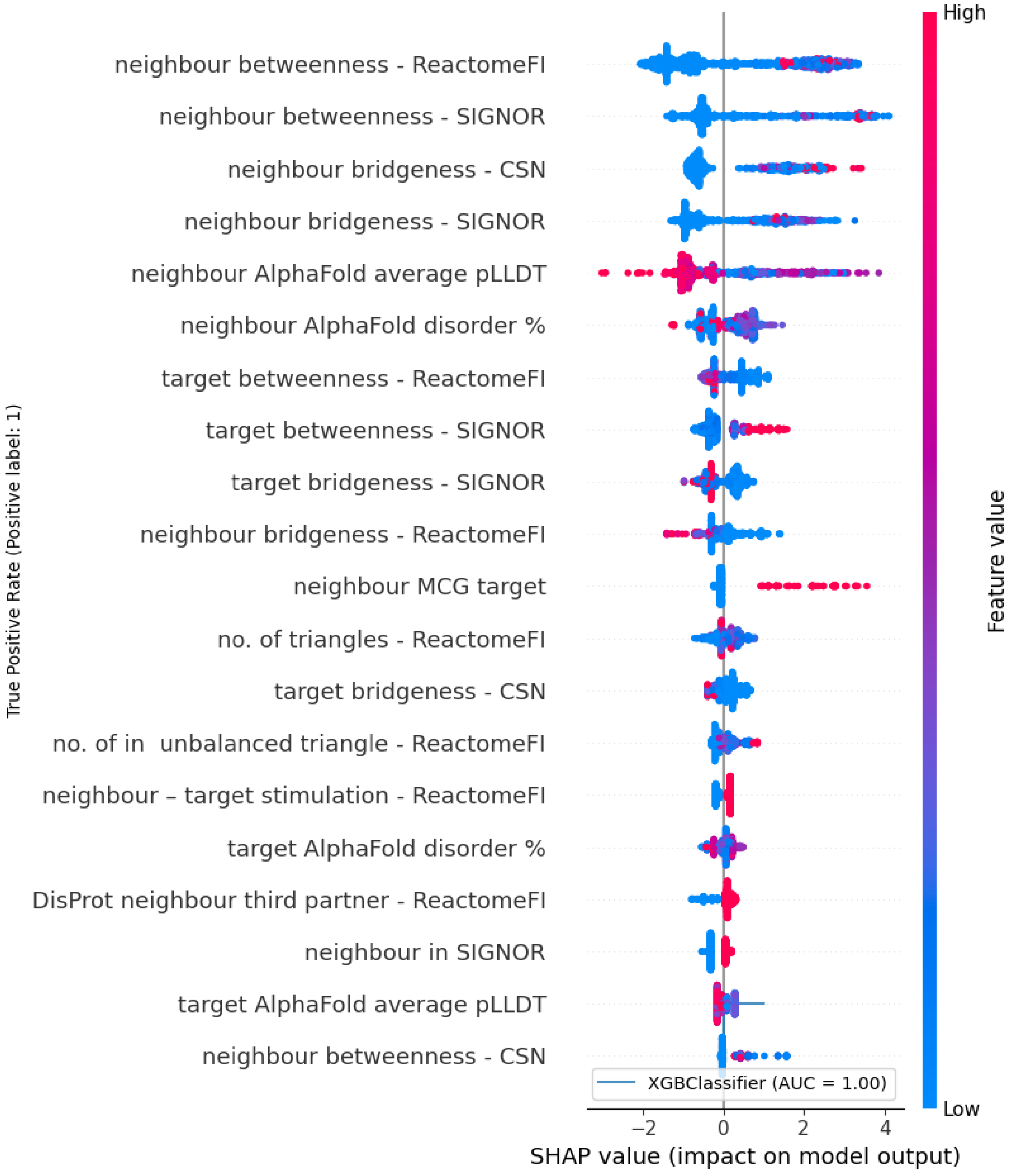

f) AlphaFold – all networks - Random Forest

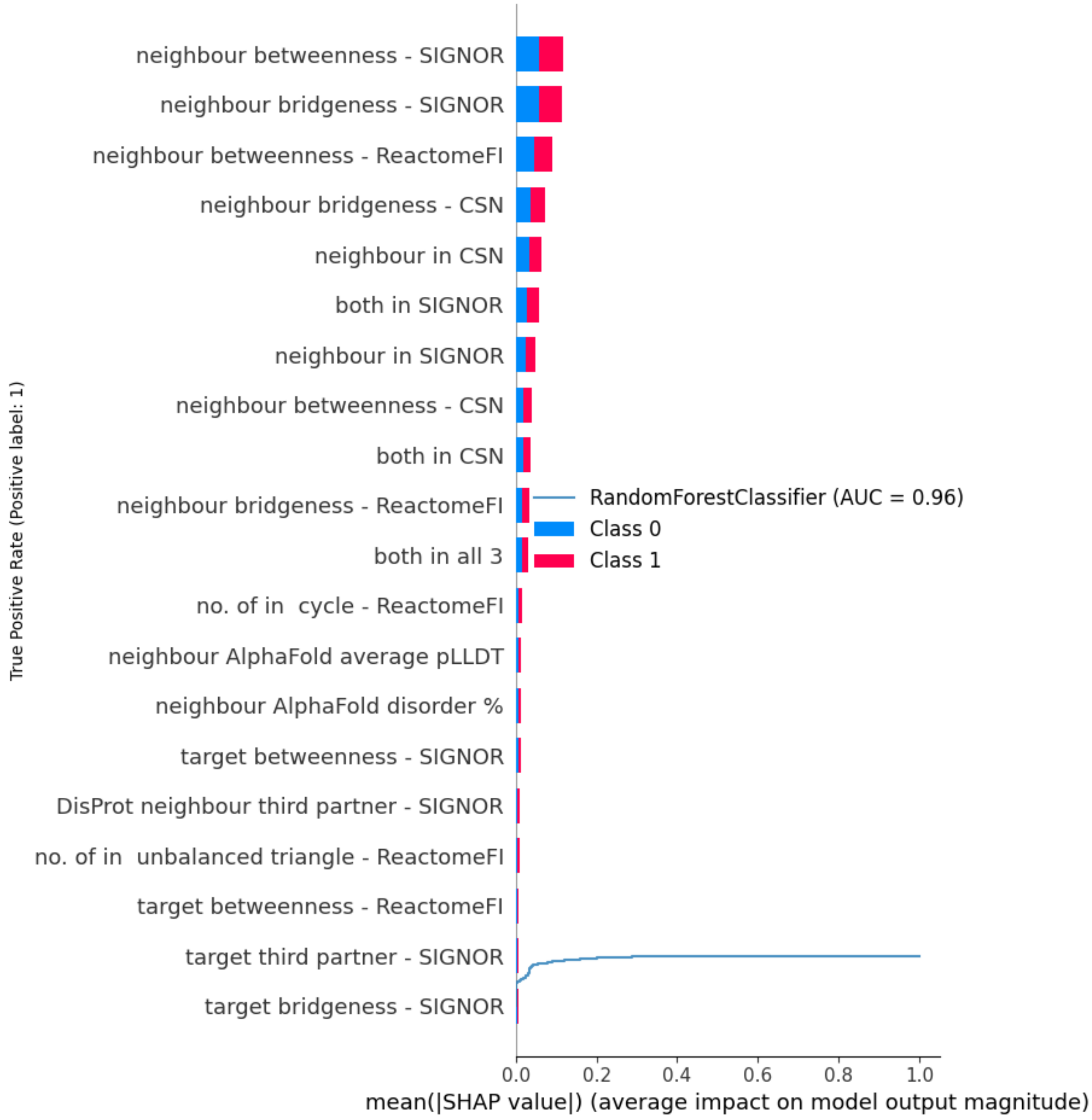

g) IUPred – all networks – XGBoost

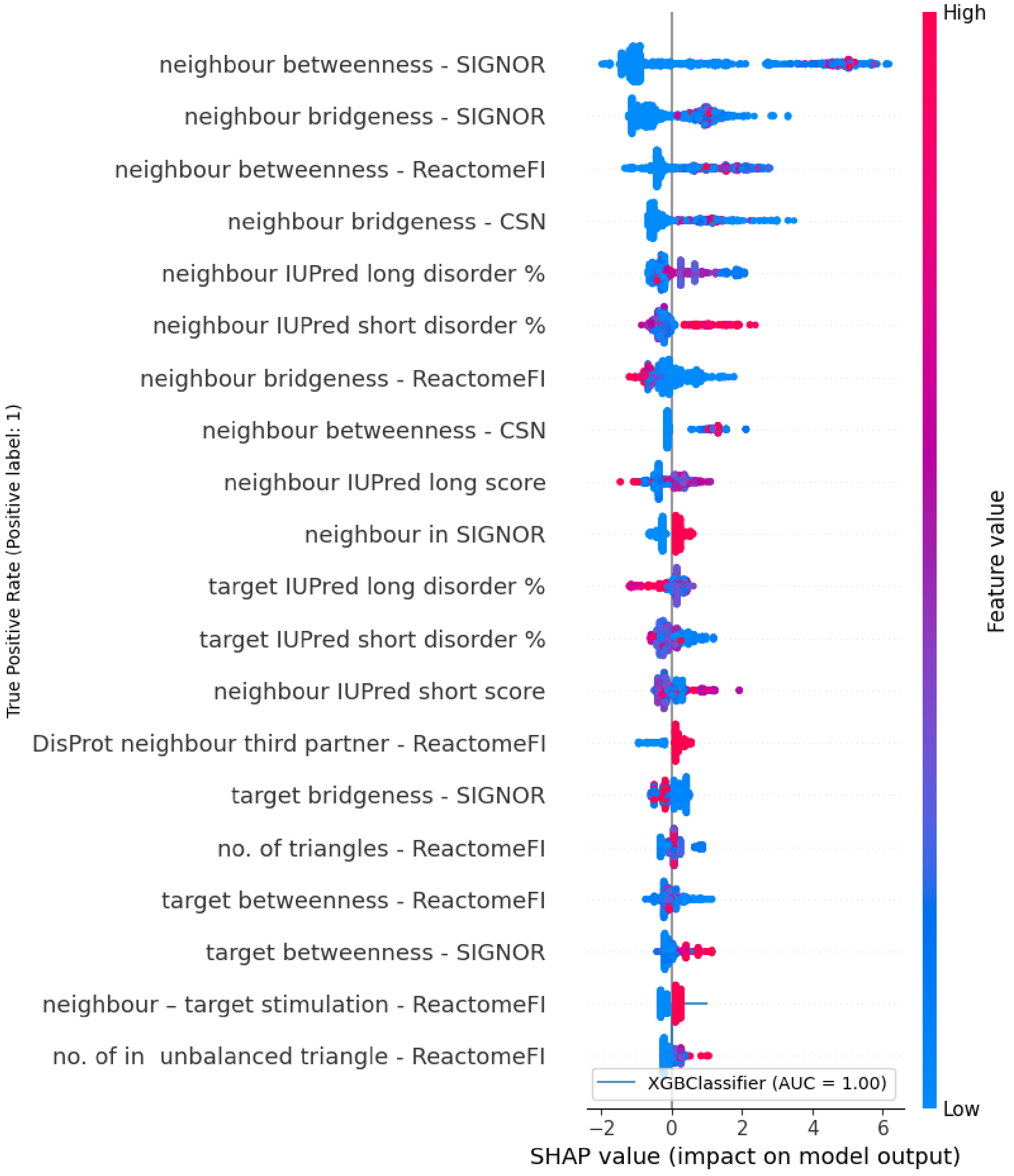

h) IUPred – all networks - Random Forest

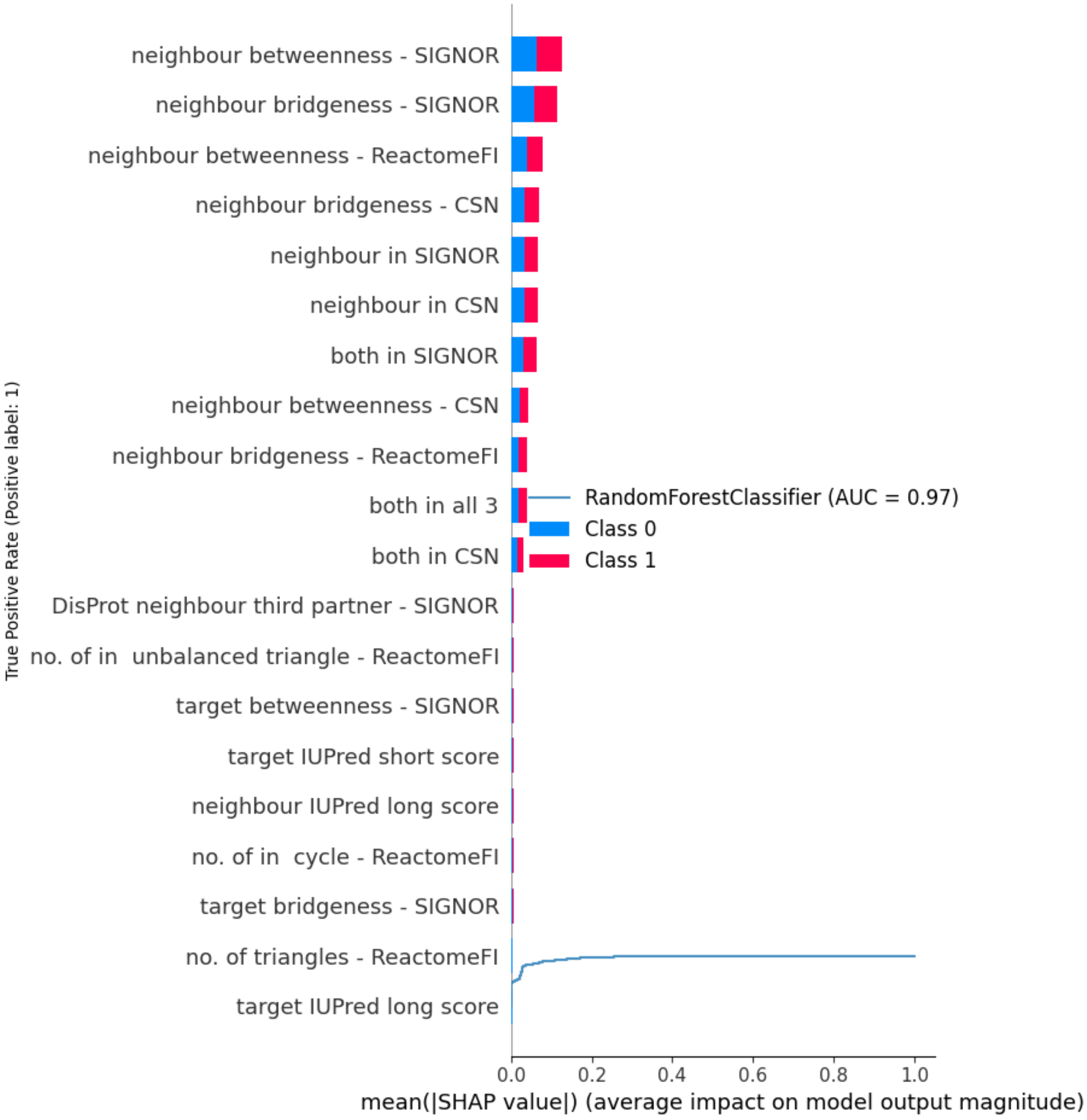

Supplementary Figure S7 | A known biomarker also predicted by the MarkerPredict framework: Notch1 as a potential predictive biomarker for HDAC-inhibitors

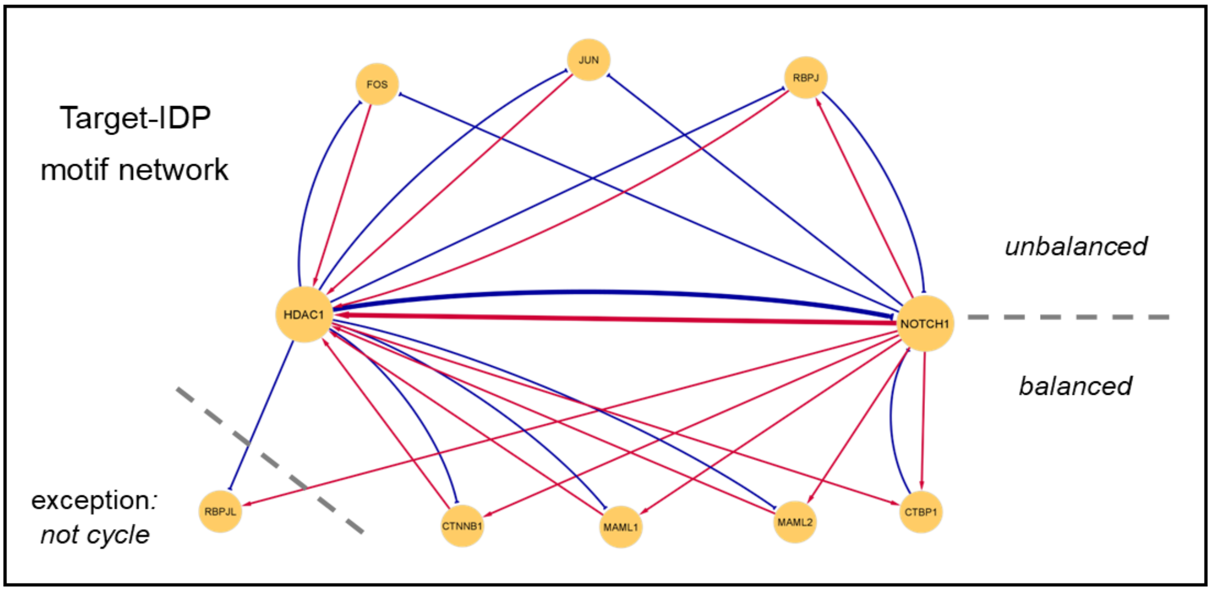

The HDAC1 and NOTCH1 interaction subnetwork in ReactomeFI. The subnetwork was visualised with the Cytoscape yFiles Organic Layout, positive and negative links were highlighted with red and blue, respectively. The eight common triangle motifs of the HDAC1-NOTCH1 pair are shown separating of the unbalanced triangles and cycles (see definition in the main text) to the top and the bottom of the figure, respectively. The negative feedback loop between HDAC1 and NOTCH1 shows the rationale behind the elevated Notch1 levels after HDAC inhibitor treatment, in regard for the regulatory effect of the adjacent regulatory motifs.

Notch1 expression is suppressed by HDAC, which is recruited by various co-repressors[3]. This regulation is modelled in the ReactomeFI network as a Notch1-HDAC feedback loop with eight different motifs. Notch1 was predicted by our method to be a potential predictive biomarker of HDAC and its inhibitors, such as vorinostat, belinostat and panobinostat. The average

Biomarker Prediction Score (BPS) of the HDAC1-Notch1 pair was 0.79, with all models classifying it as a pair with a potential predictive biomarker.

In pheochromocytoma tumour cells, HDAC inhibition resulted in elevated Notch1 levels through the transcriptional mechanism[4]. A clinical trial for panobinostat in neuroendocrine tumours suggests Notch1 as a potential predictive biomarker for this therapy, however, the regular clinical application is still to come[5]. Nevertheless, it can be showed that the MarkerPredict method was able to predict a potential predictive biomarker whose expression level is indeed closely related to the effect of the therapy used.

Supplementary Figure S8 | A new biomarker predicted by the MarkerPredict framework: β-catenin as a potential predictive biomarker for ponatinib

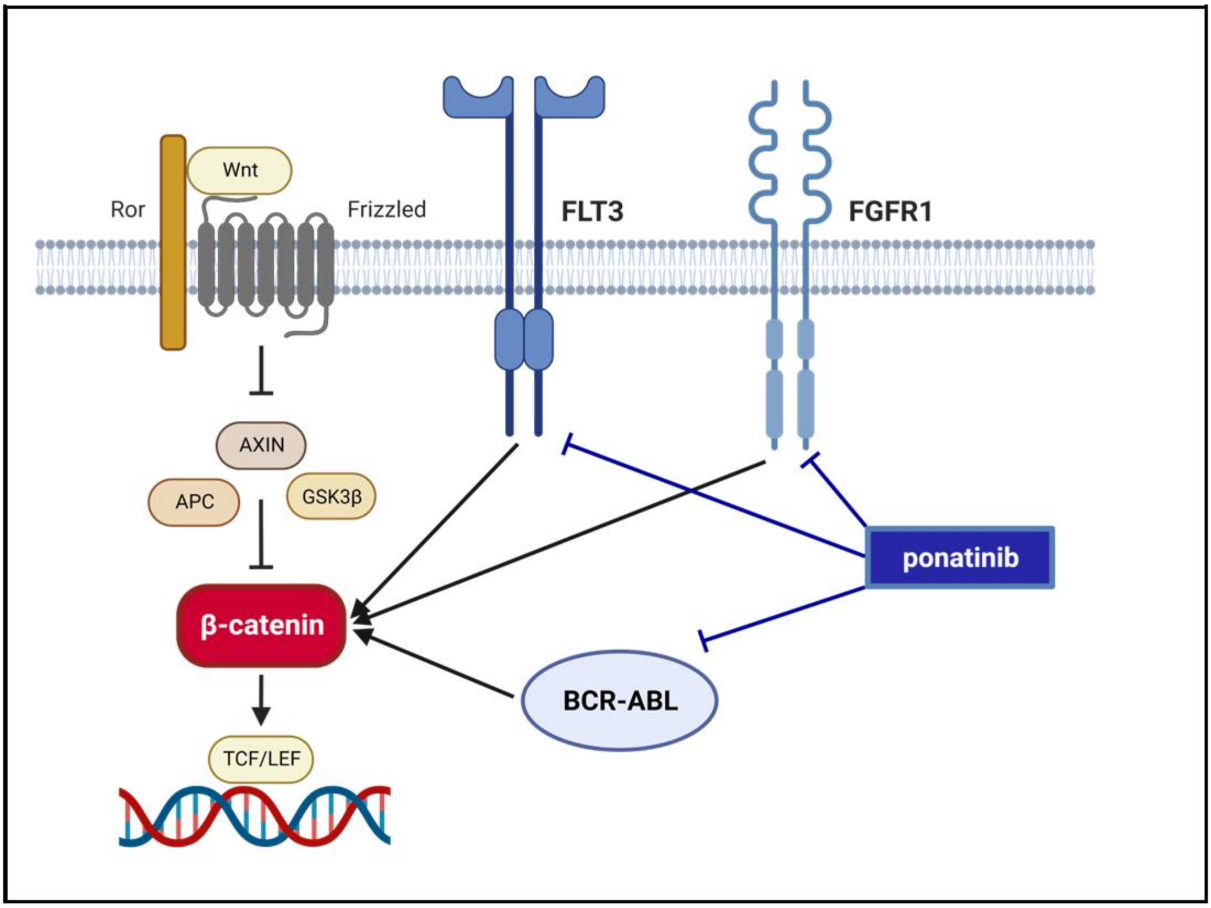

The Wnt-β-catenin network and its interaction with the targets of ponatinib. BCR-ABL, FGFR1 (ReactomeFI[6]) and FLT3 (SIGNOR[7]) phosphorylates, thus stimulates β-catenin, the product of the CTNNB1 gene, which is an IDP. Ponatinib inhibits β-catenin phosphorylation with the inhibition of these proteins. A table containing the pairs of the β-catenin IDP and the three targets of ponatinib, BCR-ABL (ABL1), FGFR1 and FLT3, the corresponding triangles and the Biomarker Probability Score (BPS) predictions are indicated, showing the strength of this prediction.

Ponatinib is a multikinase-inhibitor used in chronic myeloid leukaemia (CML) and Philadelphia chromosome positive acute lymphoblastic leukaemia (Ph+ALL) targeting the BCR-ABL fusion proteins, FGFR receptors, FLT3 and VEGFR. For its main target, BCR-ABL, the IC_{50} value is 0.37 nM[8], while for FGFR signalling (<40 nM)[9], and in mutated FLT3 (4 nM)[9] it was larger.

Ponatinib is given to patients with BCR-ABL1^{T315I} mutation causing imatinib resistance[10]. However, additional BCR-ABL mutations can cause consecutive ponatinib resistance, while in other patients, the cause of the resistance is probably the activation of other BCR-ABL independent signaling pathways[11]. The CTNNB1 gene is also often mutated in CML[12], and its overexpression may contribute to tyrosine kinase inhibitor resistance.

As fusion proteins were not considered in the analysis, the β-catenin-ABL1 pair was subjected to the *MarkerPredict* analysis, achieving a BPS of 0.89. The β-catenin-FGFR1 pair was also predicted to be a predictive biomarker-target pair, reaching a BPS score of 0.821 (see Figure). In the three pairs with all eight different models, β-catenin was classified as a potential predictive biomarker for ponatinib.

β-catenin expression is regulated through the Wnt pathway, which closely interacts with FGFR1[13]. Besides, BCR-ABL stabilizes β-catenin in CML cells with phosphorylation[14]. Additionally, in imatinib-resistant mast cells, ponatinib abrogated the phosphorylation of β-catenin at the site Y654, suppressing its translocation, and inhibiting the expression of its targets[15]. Silencing β-catenin in these cells potentiated ponatinib to induce apoptosis. However, no literature data suggests yet β-catenin as a predictive biomarker for ponatinib. Here we suggest further studying its biomarker properties based on our prediction and supporting literature data, as an additional effective biomarker for ponatinib could contribute to effective therapeutic decision-making.

Supplementary Figure S9 | A known biomarker also predicted by the MarkerPredict framework: CREB1 as a potential predictive biomarker for HER2-inhibitors

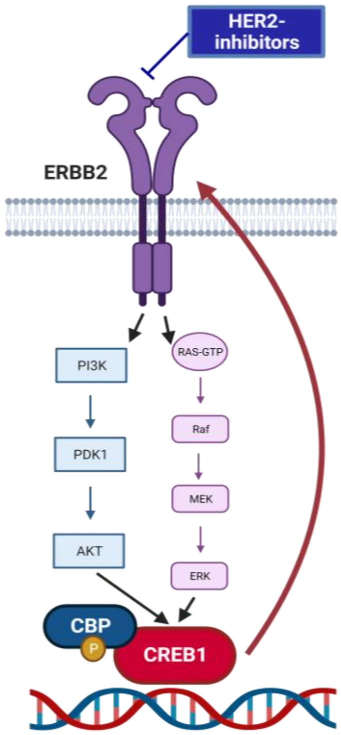

The top prediction with the highest BPS score is CREB1 as a potential predictive biomarker for HER2 (ERBB2) inhibitors. This IDP-target pair has a BPS score of 0.872, with all predictions having a probability over 0.855. In ReactomeFI network, CREB1 stimulates HER2, and they form 25 triangles together. CREB1 was defined as a prognostic, predisposing, diagnostic and predictive biomarker in the CIViCmine database[16] at the time of data collection (version 38, released July 11, 2022). HER2 is also an intrinsically disordered protein, with a disorder content of 21.35%. HER2 has several small molecule and antibody drugs targeting it, with the first of them being trastuzumab[17]. CREB1 is a transcription factor binding to the cAMP responsible

element (CRE) in the promoter regions of the DNA after getting activated by phosphorylation[18]. CREB1 is at the end of multiple signalling processes, such as the MAPK cascade, Ca^{2+} influx, and cAMP/PKA pathways[19].

Supplementary Figure S10 | A new biomarker predicted by the MarkerPredict framework: Integrin β1 as a potential predictive biomarker for c-Met inhibitor cabozantinib

The strongest novel prediction of MarkerPredict is that ITGB1, i.e., integrin β1 may be a predictive biomarker for MET inhibitors, such as cabozantinib and crizotinib. The average BPS score of this IDP-target pair is 0.905. The values were all above 0.833, thus the models confidently classified ITGB1 as a potential predictive biomarker for MET (class 1). In the ReactomeFI network, ITGB1 stimulates MET, and they participate in 23 triangles together, while both ITGB1 and MET has non-zero centrality values in all three networks. ITGB1 is part of the CIViCmine database as a prognostic, predisposing and diagnostic biomarker[16]. MET, which is the receptor of the hepatocyte growth factor (HGF), is an IDP itself, with a disorder content of 1.44%[20]. These factors could all contribute to the models' high probability values, thus to the high BPS score.

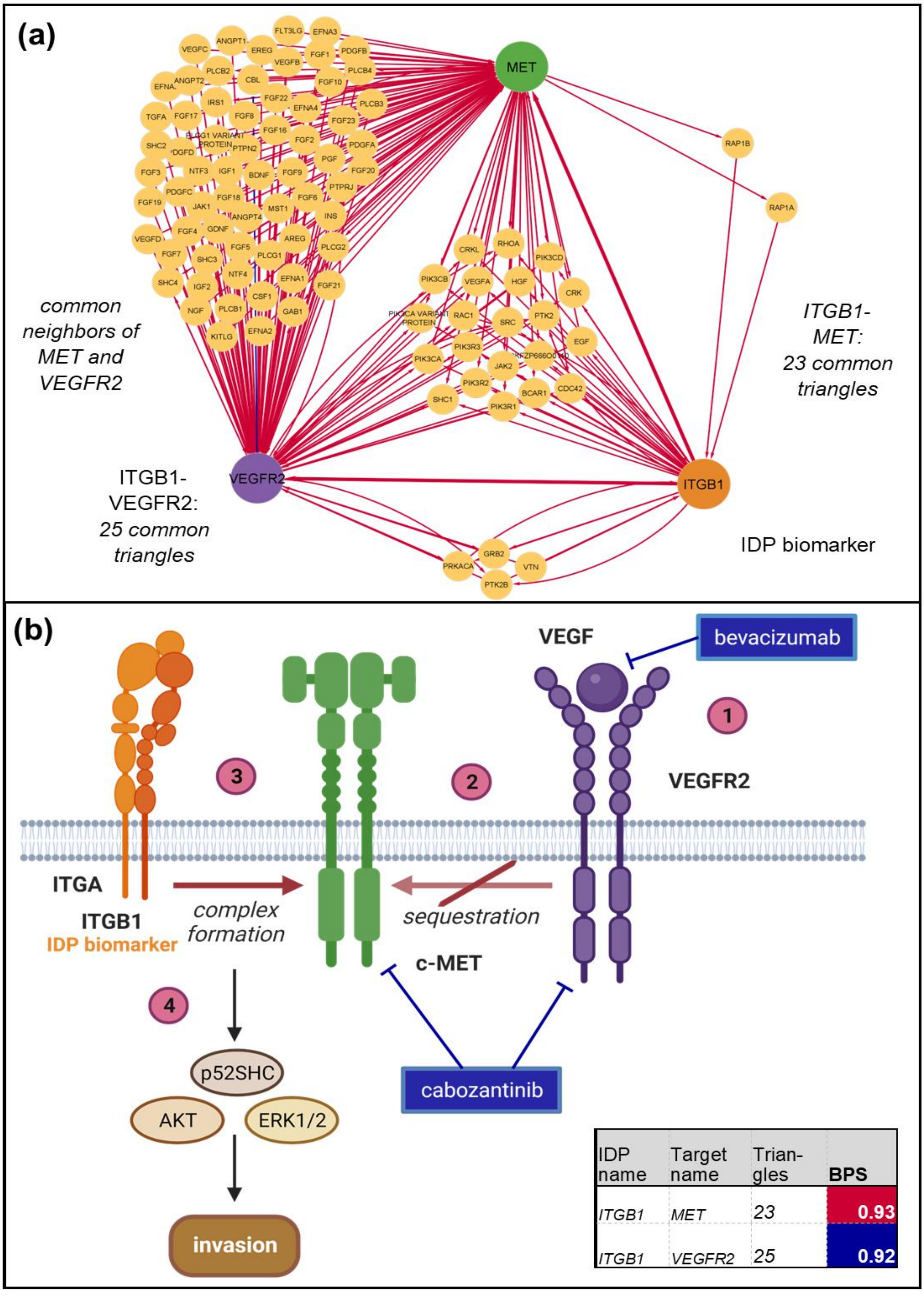

IDP name	Target name	Trian-gles	BPS
ITGB1	*MET*	*23*	0.93
ITGB1	*VEGFR2*	*25*	0.92

Figure 1 | The predicted biomarkers with the highest Biomarker Probability Score (BPS). (a) Network motifs containing the ITGB1-MET and ITGB1-VEGFR2 IDP-target pair, and the common neighbours of MET and VEGFR2 in the ReactomeFI network. The IDP target pairs had 23 and 25 motifs, respectively. Out of them, 21 nodes were in connection with both MET, VEGFR2 and ITGB1. Despite many nodes being in connection with MET and VEGFR2, their connection described in literature was not represented in our networks. (b) The ITGB1-c-MET complex formation induces invasion under bevacizumab, thus causing therapy resistance[20]. 1) Bevacizumab blocks VEGFR2 (KDR) activation by inhibiting VEGF. 2) The inactive VEGFR2 cannot sequester c-MET. 3) The not sequestered c-MET and integrins including ITGB1 can form complexes. 4) The new complex activates signalling pathways that lead to invasion and metastasis formation, causing therapy resistance to bevacizumab. Thus, the expression level or mutation of ITGB1 can show the probability of the occurring of this resistance mechanism. Cabozantinib inhibits both VEGFR2 and c-MET, thus can be a good candidate to treat the occurring therapy resistance[21]. Both the ITGB1-MET and the ITGB1-VEGFR2 IDP-target pairs were included in our networks, and they belonged to the unlabelled dataset used for the final prediction. The resulting BPS values are highlighted in the right corner of the figure.

Supplementary Table S1 | Topological parameters of the three signalling networks used in the study

The topological parameters of the three analysed signalling networks Human Cancer Signalling Network (HCN network)[21], SIGNOR[7] and ReactomeFI[6]. Number of nodes and edges apply after isolating the signed subnetwork in each case. Network density was calculated from the number of nodes and edges with the equation $\eta=2|E|/(|V|(|V|-1))$. The number of triangles was determined with the FANMOD program[22] (see Methods).

**Network**	**CSN**	**SIGNOR**	**ReactomeFI**
No. of nodes	*1 229*	*4 486*	*110 600*
No. of edges	*3 144*	*10 935*	*128 658*
Network density	*0.417*	*0.109*	*0.002*
No. of triangles	*976*	*2318*	*840 505*

Supplementary Table S2 | The input dataset of the machine learning model *(available on GitHub)*

a) The input datasets for the machine learning models, containing all the network topological data and annotations can be found on GitHub as a .csv file (as part of the MarkerPredict package, see the GitHub link at Supplementary Text S2). The preparation of the database- and network-specific training dataset is included in the code. The positive training set consists of pairs when the neighbour is a biomarker for the drug targeting the target. The negative training set was defined as pairs where the neighbour was not present in the CIViCmine database as any type of biomarker. Randomly selected pairs were added to this for the DisProt database, as otherwise the negative training set would have been too small. Complete input file of annotated neighbour-target pairs *(all_pairs.csv on GitHub)*

b) The simplified list of the neighbour-target pairs:

Supplementary Table S3 | The input parameters of the machine learning model

The input parameters for the machine learning models, including all the network topological data and annotations for multiple databases and networks.

neighbour proper-ties	target proper-ties	in network	in triangles	link type	third neughbour	centra-lities
neighbour in CSN	target in CSN	both in CSN	in triangle - CSN	neighbour – target inhibition - CSN	DisProt neighbour third partner - CSN	neighbour bridgeness - CSN

neighbour in SIGNOR	target in SIGNOR	both in SIGNOR	in triangle - SIGNOR	target - neighbour stimulation - CSN	target third partner - CSN	neighbour betweeness - CSN
neighbour in ReactomeFI	target in ReactomeFI	both in ReactomeFI	in triangle - ReactomeFI	neighbour - target stimulation - CSN	DisProt neighbour third partner - SIGNOR	target bridgeness - CSN
neighbour DisProt IDP	target of small molecule	both in all 3	not in any triangle	target - neighbour inhibition - CSN	target third partner - SIGNOR	target betweeness - CSN
neighbour DisProt disorder content	target of antibody		no. of triangles - CSN	neighbour - target inhibition - SIGNOR	DisProt neighbour third partner - ReactomeFI	neighbour bridgeness - SIGNOR
neighbour in AlphaFold	target DisProt IDP		no. of triangles - SIGNOR	target - neighbour stimulation - SIGNOR	target third partner - ReactomeFI	neighbour betweeness - SIGNOR
neighbour AlphaFold average pLLDT	target DisProt disorder content		no. of triangles - ReactomeFI	neighbour - target stimulation - SIGNOR		target bridgeness - SIGNOR
neighbour AlphaFold disorder %	target AlphaFold average pLLDT		no. of in cycle - CSN	target - neighbour inhibition - SIGNOR		target betweeness - SIGNOR
neighbour in IUPred	target AlphaFold disorder %		no. of in cycle - SIGNOR	neighbour - target inhibition - ReactomeFI		neighbour bridgeness - ReactomeFI
neighbour IUPred long score	target IUPred long score		no. of in cycle - ReactomeFI	target - neighbour stimulation - ReactomeFI		neighbour betweeness - ReactomeFI
neighbour IUPred long disorder %	target IUPred long disorder %		no. of in unbalanced triangle - CSN	neighbour - target stimulation - ReactomeFI		target bridgeness - ReactomeFI
neighbour IUPred short score	target IUPred short score		no. of in unbalanced triangle - SIGNOR	target - neighbour inhibition - ReactomeFI		target betweeness - ReactomeFI
neighbour IUPred short disorder %	target IUPred short disorder %		no. of in unbalanced triangle - ReactomeFI			

**neighbour preclinical target**						
**neighbour MCG target**						

Supplementary Table S4 | Performance of the machine learning models with different validation methods

Performance of the Random Forest and XGBoost models with different validation metrics. Twenty-four different network-specific, database-specific and combined models were trained for each validation scenario against a positive and CIViCmine-defined non-biomarker negative control set (for DisProt, random pairs were added to the negative control, see *Methods*). Sensitivity, specificity, accuracy, F1-score and the area under curve (AUC) of the receiver operating characteristic (ROC) curve were calculated for the models with 70:30 split of the input data, while average ROC AUC and accuracy for the ten-fold cross-validation and leave-one-out-cross-validation (LOOCV), respectively. Generally, all models performed well, although the XGBoost algorithm has a marginally higher performance.

**Database**	**Network**	**Model**	**70:30 split**					**10-fold cross-validation**	**LOOCV**
			Sensitivity	***Specificity***	***Accuracy***	***F1-score***	***ROC AUC***	***Average ROC AUC***	***Average accuracy***
**All Databases**	Combined	*XGBoost*	0.92	0.98	0.95	0.94	0.95	0.98	0.96
		Random Forest	0.86	0.92	0.9	0.86	0.89	0.96	0.9
	CSN	*XGBoost*	0.86	0.98	0.94	0.91	0.92	0.98	0.95
		Random Forest	0.81	0.95	0.9	0.86	0.88	0.93	0.86
	SIGNOR	*XGBoost*	0.90	0.97	0.94	0.92	0.93	0.98	0.96
		Random Forest	0.93	0.90	0.91	0.88	0.91	0.96	0.89
	ReactomeFI	*XGBoost*	0.88	0.99	0.95	0.92	0.93	0.98	0.96
		Random Forest	0.84	0.93	0.9	0.86	0.89	0.94	0.89
**DisProt**	Combined	*XGBoost*	0.68	0.87	0.77	0.76	0.77	0.84	0.77

		Random Forest	0.72	0.80	0.76	0.76	0.76	0.8	0.72
	CSN	*XGBoost*	0.68	0.80	0.74	0.73	0.74	0.82	0.77
		Random Forest	0.72	0.70	0.71	0.72	0.71	0.74	0.7
	SIGNOR	*XGBoost*	0.74	0.93	0.83	0.82	0.84	0.83	0.81
		Random Forest	0.60	0.80	0.7	0.67	0.7	0.79	0.72
	ReactomeFI	*XGBoost*	0.62	0.85	0.73	0.7	0.73	0.85	0.74
		Random Forest	0.74	0.85	0.79	0.79	0.79	0.78	0.74
**AlphaFold**	Combined	*XGBoost*	0.94	0.99	0.97	0.96	0.96	0.98	0.95
		Random Forest	0.89	0.93	0.92	0.9	0.91	0.95	0.89
	CSN	*XGBoost*	0.90	0.97	0.94	0.93	0.94	0.97	0.92
		Random Forest	0.82	0.93	0.88	0.86	0.87	0.89	0.84
	SIGNOR	*XGBoost*	0.92	0.94	0.93	0.92	0.93	0.98	0.93

		Random Forest	0.87	0.88	0.88	0.87	0.88	0.95	0.87
	ReactomeFI	*XGBoost*	0.92	0.97	0.95	0.94	0.95	0.98	0.94
		Random Forest	0.83	0.92	0.88	0.86	0.88	0.93	0.86
**IUPred**	Combined	*XGBoost*	0.90	0.98	0.95	0.93	0.94	0.98	0.96
		Random Forest	0.87	0.88	0.88	0.84	0.88	0.96	0.9
	CSN	*XGBoost*	0.89	0.96	0.94	0.91	0.93	0.97	0.93
		Random Forest	0.80	0.90	0.86	0.81	0.85	0.9	0.86
	SIGNOR	*XGBoost*	0.92	0.98	0.95	0.94	0.95	0.98	0.96
		Random Forest	0.91	0.85	0.87	0.84	0.88	0.95	0.88
	ReactomeFI	*XGBoost*	0.89	0.99	0.95	0.93	0.94	0.98	0.95
		Random Forest	0.80	0.90	0.86	0.81	0.85	0.92	0.87

Supplementary Table S5 | The results of the final prediction *(available on GitHub)*

We list the final predictions and probability of class 1 with 32 different models, as well as the 4 calculated Biomarker Probability Score (BPS) values of the neighbour-target pairs. This table is attached in a separate Excel sheet due to its large size under *BPS_score_calculation.xlsx*.

Supplementary References

1 Pedregosa, F., V., G. & al. Scikit-learn: machine learning in Python. *Journal of Machine Learning Research* **12**, 2825-2830 (2011).

2 Lundberg, S. M. & Lee, S.-I. A unified approach to interpreting model predictions. *31st Conference on Neural Information Processing Systems (NIPS 2017)*, pp. 4768 - 4777 (2017).

3 Zanotti, S. & Canalis, E. Notch and the skeleton. *Mol Cell Biol* **30**, 886-896 (2010). https://doi.org/10.1128/MCB.01285-09

4 Adler, J. T., Hottinger, D. G., Kunnimalaiyaan, M. & Chen, H. Histone deacetylase inhibitors upregulate Notch-1 and inhibit growth in pheochromocytoma cells. *Surgery* **144**, 956-961; discussion 961-952 (2008). https://doi.org/10.1016/j.surg.2008.08.027

5 Jin, N. *et al.* A phase II trial of a histone deacetylase inhibitor panobinostat in patients with low-grade neuroendocrine tumors. *Oncologist* **21**, 785-786 (2016). https://doi.org/10.1634/theoncologist.2016-0060

6 Wu, G., Dawson, E., Duong, A., Haw, R. & Stein, L. ReactomeFIViz: a Cytoscape app for pathway and network-based data analysis. *F1000Res* **3**, 146 (2014). https://doi.org/10.12688/f1000research.4431.2

7 Lo Surdo, P. *et al.* SIGNOR 3.0, the SIGnaling network open resource 3.0: 2022 update. *Nucleic Acids Res* **51**, D631-D637 (2023). https://doi.org/10.1093/nar/gkac883

8 O'Hare, T. *et al.* AP24534, a pan-BCR-ABL inhibitor for chronic myeloid leukemia, potently inhibits the T315I mutant and overcomes mutation-based resistance. *Cancer Cell* **16**, 401-412 (2009). https://doi.org/10.1016/j.ccr.2009.09.028

9 Gozgit, J. M. *et al.* Ponatinib (AP24534), a multitargeted pan-FGFR inhibitor with activity in multiple FGFR-amplified or mutated cancer models. *Mol Cancer Ther* **11**, 690-699 (2012). https://doi.org/10.1158/1535-7163.MCT-11-0450

10 Massaro, F., Molica, M. & Breccia, M. Ponatinib: A review of efficacy and safety. *Curr Cancer Drug Targets* **18**, 847-856 (2018). https://doi.org/10.2174/1568009617666171002142659

11 Eiring, A. M. *et al.* beta-Catenin is required for intrinsic but not extrinsic BCR-ABL1 kinase-independent resistance to tyrosine kinase inhibitors in chronic myeloid leukemia. *Leukemia* **29**, 2328-2337 (2015). https://doi.org/10.1038/leu.2015.196

12 Abdulmawjood, B., Costa, B. & al. Genetic biomarkers in chronic myeloid leukemia: What have we learned so far? *Int J Mol Sci* **22**, 12516 (2021). https://doi.org/10.3390/ijms222212516

13 Tang, D., He, Y., Li, W. & Li, H. Wnt/beta-catenin interacts with the FGF pathway to promote proliferation and regenerative cell proliferation in the zebrafish lateral line neuromast. *Exp Mol Med* **51**, 1-16 (2019). https://doi.org/10.1038/s12276-019-0247-x

14 Coluccia, A. M. *et al.* Bcr-Abl stabilizes beta-catenin in chronic myeloid leukemia through its tyrosine phosphorylation. *EMBO J* **26**, 1456-1466 (2007). https://doi.org/10.1038/sj.emboj.7601485

15 Jin, B., Ding, K. & Pan, J. Ponatinib induces apoptosis in imatinib-resistant human mast cells by dephosphorylating mutant D816V KIT and silencing beta-catenin signaling.

Mol Cancer Ther **13**, 1217-1230 (2014). https://doi.org/10.1158/1535-7163.MCT-13-0397

16 Lever, J. *et al.* Text-mining clinically relevant cancer biomarkers for curation into the CIViC database. *Genome Med* **11**, 78 (2019). https://doi.org/10.1186/s13073-019-0686-y

17 Ali Naderi, Chia, K. M. & Liu, J. Synergy between inhibitors of androgen receptor and MEK has therapeutic implications in estrogen receptor-negative breast cancer. *Breast Cancer Res* **13**, R36 (2011).

18 Berkowitz, L. A. & Gilman, M. Z. Two distinct forms of active transcription factor CREB (cAMP response element binding protein). *Proc Natl Acad Sci U S A* **87**, 5258-5262 (1990). https://doi.org/10.1073/pnas.87.14.5258

19 Wang, H., Xu, J., Lazarovici, P., Quirion, R. & Zheng, W. cAMP Response Element-Binding Protein (CREB): A possible signaling molecule link in the pathophysiology of schizophrenia. *Front Mol Neurosci* **11**, 255 (2018). https://doi.org/10.3389/fnmol.2018.00255

20 Quaglia, F. *et al.* DisProt in 2022: improved quality and accessibility of protein intrinsic disorder annotation. *Nucleic Acids Res* **50**, D480-D487 (2022). https://doi.org/10.1093/nar/gkab1082

21 Cui, Q. *et al.* A map of human cancer signaling. *Mol Syst Biol* **3**, 152 (2007). https://doi.org/10.1038/msb4100200

22 Wernicke, S. & Rasche, F. FANMOD: a tool for fast network motif detection. *Bioinformatics* **22**, 1152-1153 (2006). https://doi.org/10.1093/bioinformatics/btl038
